# Structure-guided unlocking of Na_X_ reveals a non-selective tetrodotoxin-sensitive cation channel

**DOI:** 10.1038/s41467-022-28984-4

**Published:** 2022-03-17

**Authors:** Cameron L. Noland, Han Chow Chua, Marc Kschonsak, Stephanie Andrea Heusser, Nina Braun, Timothy Chang, Christine Tam, Jia Tang, Christopher P. Arthur, Claudio Ciferri, Stephan Alexander Pless, Jian Payandeh

**Affiliations:** 1 Department of Structural Biology, Genentech Inc., South San Francisco, CA 94080 USA; 2 Department of Drug Design and Pharmacology, University of Copenhagen, Copenhagen, DK 2100 Denmark; 3 Department of BioMolecular Resources, Genentech Inc., South San Francisco, CA 94080 USA; 4 Department of Microchemistry, Proteomics & Lipidomics, Genentech Inc., South San Francisco, CA 94080 USA; 5 Present Address: Department of Computational and Structural Chemistry, Merck Research Labs, South San Francisco, CA 94080 USA; 6 Present Address: Department of Drug Discovery Sciences, Boehringer Ingelheim RCV, Vienna, AU 1121 Austria; 7 Present Address: Department of Proteomics and Bioinformatics, Interline Therapeutics, South San Francisco, CA 94080 USA

**Keywords:** Electron microscopy, Physiology, Sodium channels

## Abstract

Unlike classical voltage-gated sodium (Na_V_) channels, Na_X_ has been characterized as a voltage-insensitive, tetrodotoxin-resistant, sodium (Na^+^)-activated channel involved in regulating Na^+^ homeostasis. However, Na_X_ remains refractory to functional characterization in traditional heterologous systems. Here, to gain insight into its atypical physiology, we determine structures of the human Na_X_ channel in complex with the auxiliary β3-subunit. Na_X_ reveals structural alterations within the selectivity filter, voltage sensor-like domains, and pore module. We do not identify an extracellular Na^+^-sensor or any evidence for a Na^+^-based activation mechanism in Na_X_. Instead, the S6-gate remains closed, membrane lipids fill the central cavity, and the domain III-IV linker restricts S6-dilation. We use protein engineering to identify three pore-wetting mutations targeting the hydrophobic S6-gate that unlock a robust voltage-insensitive leak conductance. This constitutively active Na_X_-QTT channel construct is non-selective among monovalent cations, inhibited by extracellular calcium, and sensitive to classical Na_V_ channel blockers, including tetrodotoxin. Our findings highlight a functional diversity across the Na_V_ channel scaffold, reshape our understanding of Na_X_ physiology, and provide a template to demystify recalcitrant ion channels.

## Abstract (web summary)

Na_X_ is an atypical member of the voltage-gated sodium channel family that may contribute to Na^+^ homeostasis. Here, the authors describe the structural and functional attributes of the human Na_X_ channel to reveal new insights into its physiology.

## Introduction

Mammalian voltage-gated sodium (Na_V_) channel subtypes, Na_V_1.1–Na_V_1.9, perform essential roles in electrical signaling by initiating and propagating action potentials^1^. A tenth Na_V_ channel-like gene (*SCN7A*) was cloned three decades ago, but atypical sequence features and the inability to record voltage-activated sodium (Na^+^)-currents raised speculation that this channel might have distinct physiological roles^2–5^. Evidence suggests that Na_X_ may contribute to Na^+^ homeostasis^5–10^ and Na_X_ shows restricted expression in a brain area that specializes in monitoring blood composition^11–13^. Na_X_-knockout mice ingest salt despite dehydration and are resistant to hypertension caused by elevated Na^+^ levels^11,14^. Autoimmunity against Na_X_ is associated with chronic hypernatremia with impaired thirst perception and salt appetite in humans^9^. In the periphery, Na_X_ regulates Na^+^ homeostasis upstream of the epithelial Na^+^ channel (ENaC) and may be a target to treat atopic dermatitis and hypertrophic scarring^10,15^.

Clues to Na_X_ function derive mainly from in vivo studies and cells obtained from knockout mice^5,16^. Murine Na_X_ is reportedly activated by extracellular Na^+^ concentrations above 150 mM^5,17,18^, where Na^+^-sensitivity can be shifted into the physiological range (135–145 mM) through a mechanism involving the endothelin receptor^17^. Na^+^ influx through Na_X_ may activate the Na^+^/K^+^-ATPase^19,20^ leading to proton release and the activation of acid-sensing channels on nearby neurons^14,16^. However, because all attempts to record currents from traditional heterologous systems have failed^3,4^, the basic biophysical properties of Na_X_ and the molecular determinants involved in Na^+^-sensing remain unknown.

Na_X_ is the most sequence divergent member of the mammalian Na_V_ channel family^1^, which has made structure-function correlations difficult to discern. Because murine Na_X_ contains a reduced number of S4-gating charges and is voltage-insensitive^2–4^, the structure and function of the voltage-sensor-like domains (VSLDs) are unclear. Instead of the characteristic ion-selectivity filter DEKA-locus found in Na_V_ channels, Na_X_ contains a unique DENA-locus^2–4^ and is thought to be tetrodotoxin (TTX)-resistant^5,12^. Na_X_ is reported to generate non-inactivating currents and has a divergent intracellular DIII–DIV linker sequence^5,18^. In this work, we determined the structure of the human Na_X_ channel, which has provided new insights into its unusual biophysical properties and physiology.

## Results

### Evaluation of human Na_X_ function

**Fig. 1:**
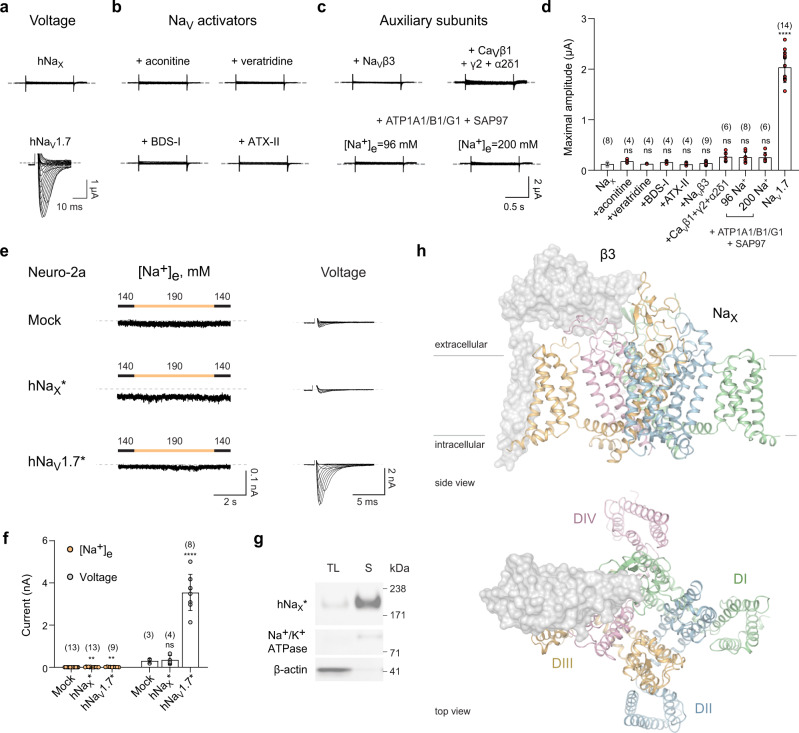
Characterization of and overall structure of human Na_X_. **a** Representative currents from *Xenopus laevis* oocytes expressing human Na_X_ or Na_V_1.7. Na_X_: steps between +80 to −100 mV, in 20 mV increments from a HP of 0 mV; Na_V_1.7: depolarizing steps between −80 and +65 mV, in 5 mV increments, from a HP of −100 mV. **b** Representative currents from oocytes expressing Na_X_ in response to extracellular application of indicated compounds (BDS-I Blood depressing substance I, ATX-II Neurotoxin 2). Voltage protocols as above. See Methods for concentrations of compounds tested. **c** Representative currents from oocytes expressing Na_X_ and co-expression of Na_V_ or Ca_V_ channel auxiliary-subunits, Na^+^/K^+^-ATPase subunits and synapse-associated protein 97 (SAP97), and in the presence of the indicated extracellular Na^+^ concentration. Voltage protocols as above. **d** Data summary of independent experiments performed as in parts **a**–**c**. Data are shown as mean ± SD; ns not significant; ****p < 0.0001; one-way ANOVA with Dunnett’s test (against Na_X_). Exact *p*-values and statistical parameters are provided in Source Data. Numbers of biological replicates (*n*) are indicated. **e** Representative currents from murine Neuro-2a cells expressing human Na_X_ or Na_V_1.7 in response to changes of the extracellular Na^+^ concentration (HP = −60 mV), as indicated, or voltage: depolarizing steps between −60 to +60 mV, in 10 mV increments from a HP of −100 mV. **f** Data summary of independent experiments performed as in parts **e**. Data are shown as mean ± SD; ns not significant; *p < 0.05; **p < 0.01; ****p < 0.0001; one-way ANOVA with Dunnett’s test (against mock-transfected cells). Exact *p*-values and statistical parameters are provided in Source Data. Numbers of biological replicates (*n*) are indicated. **g** Western blots of total lysate and surface fraction of proteins extracted from Neuro-2a cells probed for the indicated proteins. Data represent three independent biological replicates. **h** Side and extracellular view of the β3-Na_X_ channel complex. Approximate membrane boundaries are indicated. DI, DII, DIII, and DIV are colored in green, blue, orange, and pink, respectively, with the β3-subunit in gray surface representation.

Reliable ionic currents were not detected for human Na_X_ expressed in HEK293T cells or *Xenopus laevis* oocytes in response to changes in membrane voltage (Fig. 1a–d, Supplementary Fig. 1a–d). Application of Na_V_ channel pharmacological activators like aconitine or veratridine did not invoke any clear Na_X_-mediated currents in oocytes (Fig. 1b, d, Supplementary Fig. 1g, h). Co-expression with Na_V_ or voltage-gated calcium (Ca_V_) channel auxiliary-subunits also failed to produce dependable currents (Fig. 1c, d, Supplementary Fig. 1i, j). Thus, as reported for the rodent proteins^3,4^, human Na_X_ does not operate as a conventional voltage-gated channel.

In a neuroblastoma (Neuro-2a) cell line expressing murine Na_X_, increases in the extracellular Na^+^ concentration ([Na^+^]_e_) reportedly produce a non-inactivating Na^+^ current^17,18^. When human Na_X_ was expressed in Neuro-2a cells, small and flickery inward currents were observed upon changes in [Na^+^]_e_ from 140 to 190 mM, but cells expressing the human Na_V_1.7 channel showed similar behavior (Fig. 1e, g, Supplementary Fig. 1e). A non-inactivating inward current was only observed when seal resistances were low (<1 GΩ) in both human Na_X_- and mock-transfected Neuro-2a cells (Supplementary Fig. 1f). Because sequence differences (Supplementary Fig. 2) or cellular context^21^ may account for these seemingly conflicting functional outcomes between murine and human Na_X_, we next considered if structure determination might provide insight into the function or regulation of Na_X_.

### Structure of the β3-Na_X_ channel complex in lipid nanodiscs

To facilitate cryogenic electron microscopy (cryo-EM) analysis, human Na_X_ was co-expressed with the canonical auxiliary β3-subunit, purified in mild detergent (glyco-diosgenin), and reconstituted into phospholipid nanodiscs (Supplementary Fig. 3a–c). Prior to sample vitrification, the Na^+^ concentration was raised above the threshold reported to activate Na_X_ (to 200 mM), and the resulting cryo-EM reconstruction of the β3-Na_X_ complex extended to 3.2 Å resolution (Supplementary Fig. 4a–g, Supplementary Table 1).

The Na_X_ channel resembles a four-leaf clover with VSLDs arranged in a domain-swapped organization (Fig. 1h). The β3-subunit is wedged between extracellular loops of the pore module and its single transmembrane helix is positioned against VSLD3 (Fig. 1h, Supplementary Fig. 5a). The intracellular DIII–DIV linker is bound alongside the pore (see below), and the carboxyl-terminal domain is not well-resolved (Supplementary Fig. 5a), establishing that the overall architecture of Na_X_ is highly similar to available human Na_V_ channel structures determined in detergent (Supplementary Fig. 5b–e)^22–26^. However, Na_X_ does reveal structural deviations in regions implicated in Na_V_ channel gating (Supplementary Fig. 5d, e), suggesting that these changes may be a consequence of sequence and functional divergence (Fig. 1a, b, Supplementary Fig. 2).

### The Na_X_ pore module is in a nonconductive state

**Fig. 2:**
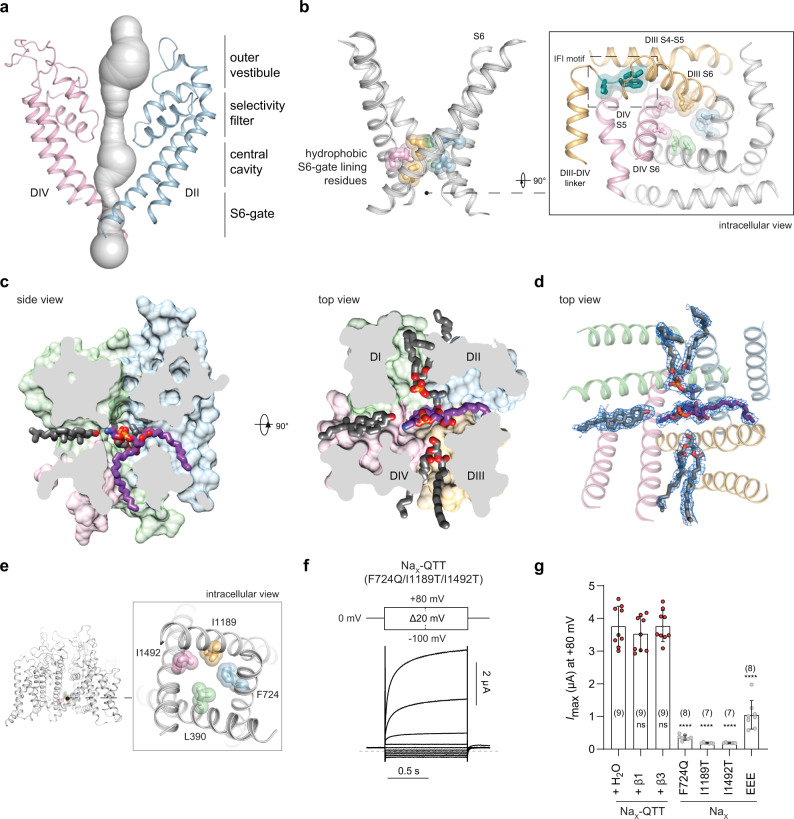
Na_X_ pore module structure reveals a nonconductive state. **a** Na_X_ pore volume shown as gray surface with DII and DIV in cartoon rendering (DI and DIII omitted for clarity). **b** View of the S6-helices with side-chains lining the activation gate shown. Orthogonal view provides a wider perspective with DIII and DIV colored orange and pink, and the IFI-motif (green) from the DIII–DIV linker shown in stick and semi-transparent surface representation. **c** Orthogonal views sliced through the pore module highlighting lateral fenestrations and bound lipids. The phosphatidylethanolamine that crosses the S6-gate is in purple stick representation. **d** Similar to middle panel **c**, but with cryo-EM map shown in blue mesh representation. **e** Location of S6-gate hydrophobic side-chains targeted by pore-wetting mutations. **f** Representative currents from *Xenopus laevis* oocytes expressing the Na_X_-QTT construct with voltage protocol indicated. **g** Data summary of independent experiments with indicated constructs (see Supplementary Fig. 8). Data are shown as mean ± SD; ns not significant; ****p < 0.0001; one-way ANOVA with Dunnett’s test (against Na_X_-QTT + H_2_O). Exact *p*-values and statistical parameters are provided in Source Data. Numbers of biological replicates (*n*) are indicated.

The Na_X_ pore module contains an outer vestibule, an ion-selectivity filter, a central cavity, and an S6-gate (Fig. 2a). Despite the high Na^+^ concentration in our sample, no distinguishing extracellular Na^+^ binding site was observed (Supplementary Fig. 5f), and residues lining the narrow S6-gate form a hydrophobic barrier to the passage of hydrated ions, defining a nonconductive state (Fig. 2b, Supplementary Fig. 5c)^22–26^. These structural observations align with our physiological results (Fig. 1a–f) and overturn expectations that human Na_X_ alone gives rise to a Na^+^-activated, non-inactivating conductance^17,18^.

The Na_X_ central cavity is similar to those in Na_V_ channels, although the DIV-S6 phenylalanine targeted by pore-blocking drugs^26,27^ is replaced by DIV-Trp1484 (Supplementary Fig. 5g). Na_X_ reveals four lateral fenestrations that penetrate the pore, suggesting that lipids or Na_V_ and Ca_V_ channel antagonists might target the central cavity through membrane access pathways (Fig. 2c, d).

### The Na_X_ pore is infiltrated by membrane lipids

Four lipids penetrate the lateral pore fenestrations in Na_X_ to enter the central cavity (Fig. 2c, d). These features can unambiguously be assigned as three phospholipids and one cholesterol, where one phospholipid even straddles and seals the intracellular S6-gate (Fig. 2c, d, Supplementary Fig. 5h). To evaluate if nanodisc reconstitution was responsible for lipid visualization, we prepared a β3-Na_X_ sample in the detergent glyco-diosgenin without exogenous phospholipid supplementation and determined its structure in 200 mM Na^+^ to 2.9 Å resolution (Supplementary Fig. 6a–g, Supplementary Table 1). An essentially identical pore structure penetrated by four well-defined lipids was observed (Supplementary Fig. 6h). The presence of well-resolved, co-purifying lipids in Na_X_ may reflect the hydrophobicity of the central cavity and S6-gate or the intrinsic stability of the nonconductive state.

### The intracellular DIII–DIV linker restricts S6-gate dilation

The Na_X_ DIII–DIV linker is bound to a pore module receptor site reminiscent of the fast-inactivated state in Na_V_ channels (Fig. 2b, Supplementary Fig. 7a, b)^22–26^. Mutating the Na_X_ IFI-motif to a Na_V_ channel non-inactivating QQQ-motif^28^ was insufficient to generate currents in oocytes (Supplementary Fig. 7c, d), but disrupting the VSLD3 and DIII S4–S5 linker regions required to form the fast-inactivation receptor site in Na_V_ channels produced robust leak currents in Na_V_1.7-Na_X_ chimeric channels (Supplementary Fig. 7e–g)^29^. The DIII–DIV linker therefore appears to restrict S6-dilation in Na_X_ and may stabilize the nonconductive state.

### Pore-wetting mutations can activate human Na_X_

We hypothesized that targeted introduction of polar residues around the hydrophobic S6-gate might promote pore hydration, destabilization of bound lipids, displacement of the IFI-motif, and transition into a conductive state (Fig. 2a–d). A triple-mutant Na_X_ channel construct containing polar substitutions at three S6-gate lining positions, Na_X_-QTT (F724Q-I1189T-I1492T), produced robust ionic currents when expressed alone in *Xenopus* oocytes (Fig. 2e, f; Supplementary Fig. 8a, b). Single-point mutant Na_X_ channels failed to produce robust currents, indicating that a pore-wetting threshold likely exists (Fig. 2g, Supplementary Fig. 8c)^30,31^. The triple-mutant construct Na_X_-EEE (L390E-I1189E-I1492E) also displayed function, but with lower current amplitudes and higher current variability relative to Na_X_-QTT (Fig. 2g, Supplementary Fig. 8d).

Na_X_-QTT currents are outward-rectifying without signs of inactivation (Fig. 2f, Supplementary Fig. 8b). Current amplitudes or kinetics were not impacted when Na_X_-QTT was co-expressed with the β1- or β3-subunits (Fig. 2g, Supplementary Fig. 8e). Na_X_-QTT also behaved as a non-inactivating channel in patch-clamp experiments in HEK293T cells (see below), where minor differences in the kinetics of the outward currents are likely due to species differences between the expression systems, and the lack of control over intracellular ionic composition in *Xenopus laevis* oocytes. Because Na_X_-QTT contains only three mutations targeted to the S6-gate (Fig. 2e), we characterized this construct as a proxy to evaluate ion selectivity and pharmacology of the human Na_X_ channel.

### The Na_X_ selectivity filter

**Fig. 3:**
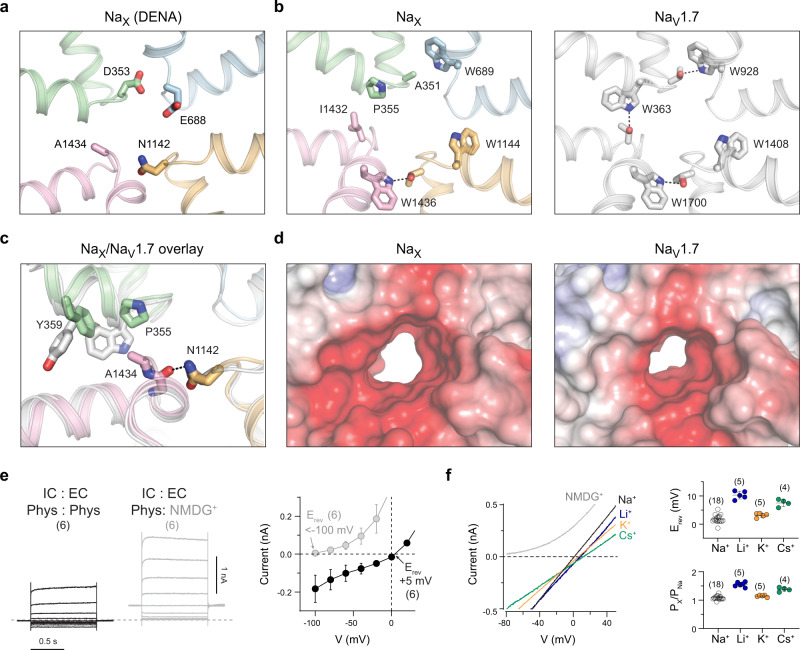
Structure and characterization of the Na_X_ selectivity filter. **a** Na_X_ DENA-motif side-chains shown as sticks. **b** Residues in Na_X_ that form a conserved interaction network around the selectivity filter in Na_V_ channels (Na_V_1.7, PDB 6J8J). **c** Superimposed view of the DI-DIV interface comparing Na_X_ and Na_V_1.7 (gray, PDB 6J8J). **d** View as in **b**, Na_X_ and Na_V_1.7 selectivity filter electrostatic surface rending. Note, central cavity and activation gate excluded for clarity. **e** Representative currents from HEK293T cells expressing human Na_X_-QTT with a C-terminal GFP-Flag tag in a physiological (left) or NMDG^+^-only extracellular solution (middle). See methods for composition of intracellular (IC) and extracellular (EC) solutions. Steps between +80 to −100 mV, in 20 mV increments, from a HP of 0 mV. Right, shows I–V curve data summary from *n* = 6 cells over two independent experiments. Data are shown as mean ± SD. Numbers of biological replicates (*n*) are indicated. **f** Representative currents from HEK293T cells expressing human Na_X_-QTT with indicated monovalent cations in the extracellular solution. Voltage ramp from −80 to +80 mV was applied. Right, summary of reversal potentials and permeability ratios measured from three independent experiments. Data are shown as mean ± SD. Numbers of biological replicates (*n*) are indicated.

The unique DENA-locus of Na_X_ is supported by a local architecture that differs radically from the scaffold which supports the DEKA-locus in Na_V_ channels (Fig. 3a–d). Consequently, the ion-selectivity filter of Na_X_ is wider and more electronegative (Fig. 3d). At the DENA-locus, Asp353 (DI) bonds to the backbone of DII-Trp689, Glu688 (DII) points into the ion permeation pathway, and Asn1142 (DIII) bonds to the DIV-Phe1433 carbonyl to impose a ~1.5 Å radial shift onto Ala1434 (DIV) relative to the DIV alanine in Na_V_ channels (Fig. 3c). Displacement of Ala1434 is accommodated by the distinct DI-Pro355 in Na_X_, which permits DI-Tyr359 to fill the volume vacated by the traditional DI tryptophan (Fig. 3b, c). This remodeling only slightly repositions side-chains within the Na_X_ outer vestibule, suggesting that ion or toxin binding above the selectivity filter may not be substantially impacted (Supplementary Fig. 9a, b).

### Na_X_-QTT is non-selective among monovalent cations

The DIII-Asn1142 dramatically alters the structure and chemical profile of the Na_X_ selectivity filter relative to the DIII-lysine that supports Na^+^-selectivity in Na_V_ channels (Fig. 3d)^32^. Transferring a DEKA-locus into Na_X_ produced no measurable currents (Supplementary Fig. 9c), establishing that features beyond the selectivity filter contribute to channel activation.

When cations in the extracellular solution were replaced with N-methyl-D-glucamine, the reversal potential for Na_X_-QTT in HEK293T cells shifted from +5 mV to below −100 mV (Fig. 3e), indicating that this construct behaves as a cation channel, and that large cations do not permeate. Replacement of Cl^−^ ions with the large anion methansulfonate did not change the current profile (Supplementary Fig. 9d), establishing that Na_X_-QTT behaves as a cation-selective channel. Using a ramp protocol with a simple Na^+^ intracellular solution (150 mM), the reversal potential was close to 0 mV under symmetrical NaCl conditions, and only slight changes in the reversal potentials were measured upon exposure to different extracellular monovalent cations (150 mM; Fig. 3f). These results demonstrate that the Na_X_-QTT selectivity filter does not effectively discriminate between Na^+^, K^+^, Cs^+^, or Li^+^ ions.

### Na_X_-QTT is inhibited by Ca^2+^ and sensitive to Na_V_ channel blockers

**Fig. 4:**
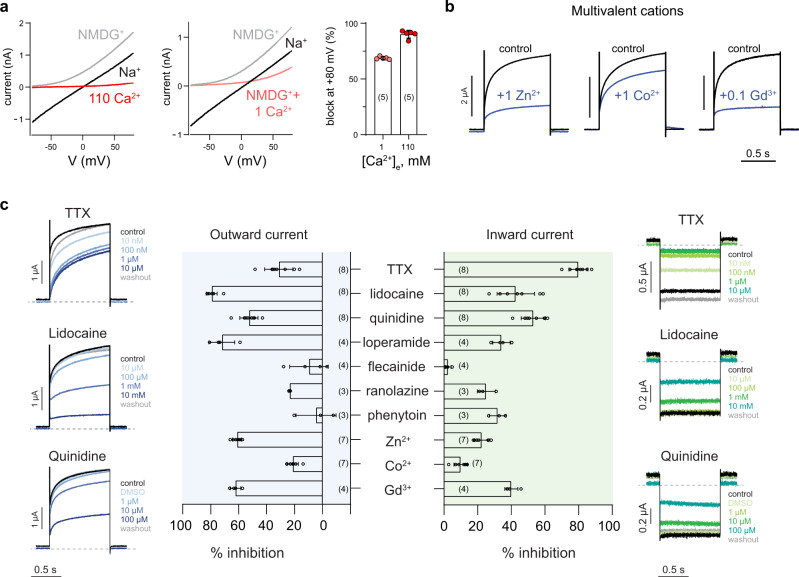
Pharmacology of the human Na_X_-QTT channel. **a** Representative I–V curves from HEK293T cells expressing human Na_X_-QTT with indicated extracellular monovalent cations with or without indicated amounts of CaCl_2_ in the extracellular solution. Voltage ramp from −80 to +80 mV was applied. Right, percentage of block of outward Na^+^ by indicated concentrations of Ca^2+^ at 80 mV. Data are shown as mean ± SD of *n* = 5 cells over three independent experiments. **b** Representative currents from *Xenopus laevis* oocytes expressing human Na_X_-QTT in standard extracellular solution with or without indicated divalent and trivalent cations (unit in mM), when stepping from 0 to +80 mV. **c** Representative currents from oocytes expressing human Na_X_-QTT in standard extracellular solution with or without indicated blockers added, when stepping from 0 to +80 mV (left) or from 0 to −100 mV (right). Middle, summary of currents measured from two independent experiments. Data are shown as mean ± SD. Numbers of biological replicates (*n*) are indicated. See Methods for concentrations of compounds tested.

Ca^2+^ does not permeate through Na_X_-QTT, but 1 mM extracellular Ca^2+^ inhibited ~70% of the Na^+^ current (Fig. 4a). Zinc (Zn^2+^), cobalt (Co^2+^), and gadolinium (Gd^3+^) cations also inhibited Na_X_-QTT currents (Fig. 4b). Contrasting long-standing reports^5,12^, tetrodotoxin showed inhibitory effects on Na_X_-QTT, especially on the inward current (Fig. 4c). The high potency of TTX-block suggests that it occurs at the selectivity filter, where most side-chains implicated in TTX-block in Na_V_ channels are structurally conserved (Supplementary Fig. 2, 9b)^24^. Lidocaine, quinidine, and loperamide blocked Na_X_-QTT currents in the µM to mM concentration range; however, strong inhibitory effects were not observed by all classical Na_V_ channel pore-blocking drugs tested (Fig. 4c).

### Na_X_ reveals atypical voltage-sensor-like domains

Conditions under which human Na_X_ channel might produce voltage-activated currents have not been identified (Fig. 1a–f), although the VSLDs share a high structural similarity with the voltage-sensor domains (VSDs) of Na_V_ and Ca_V_ channels (Supplementary Fig. 10a). Numerous features are implicated in noncanonical contributions to Na_X_ channel function, for instance, VSLD4 is found in an unusual deactivated-like state (Supplementary Fig. 10a, b).

## Discussion

The human Na_X_ channel does not function as a conventional voltage-activated channel, but we find that the transfer of entire homologous domains or subdomains between Na_X_ and Na_V_1.7 can produce channels with unusual gating characteristics (Supplementary Fig. 7d, e, 10c). These results suggest that the coupling interfaces or energetics of Na_X_ gating have diverged substantially from Na_V_ channels, underscoring the functional diversity that has evolved across this channel scaffold^33^. This does not exclude the possibility that Na_X_ may function as a voltage-modulated channel, since binding of one or more putative auxiliary proteins may impart voltage sensitivity as described for the NALCN and TMEM16A channels^33,34^.

Murine Na_X_ has been reported to produce non-inactivating inward currents in Neuro-2a cells upon increasing the extracellular Na^+^ concentration >150 mM^5,17,18^. We only observed qualitatively similar recordings with low seal resistance, and failed to reconstitute Na^+^-activated currents in cells expressing human Na_X_ (Fig. 1d, Supplementary Fig. 1f). Moreover, our β3-Na_X_ cryo-EM samples were prepared with NaCl concentrations above the reported threshold required for Na^+^-dependent gating^5,16,17^, but no clear Na^+^-sensing locus or Na^+^-based activation mechanism was observed (Fig. 2a, b, Supplementary Fig. 5f). The only map feature that we might assign as a Na^+^ ion is bound near the Na_X_ selectivity filter at an extracellular site that is conserved in Na_V_ channels (Supplementary Fig. 9a)^23^. We conclude that Na_X_ lacks an intrinsic Na^+^-sensor, and speculate that the described Na^+^-sensing mechanism must be contributed by an exogenous factor or pathway, such as the Na^+^/K^+^ ATPase, the endothelin receptor, a yet to be identified system, auxiliary protein(s), or post-translational modification^17,19,20^.

Inspired by the concept of hydrophobic gating in channels^30,31^, mutation of only three residues around the S6-gate region was sufficient to produce robust ionic currents from Na_X_-QTT in *Xenopus* oocytes and HEK293T cells (Figs. 2e, f, 3e, f, 4a–c). Conceptually, the introduced polar side-chains help to hydrate the hydrophobic S6-gate, dislodge bound membrane lipids, and displace the IFI-motif to promote pore dilation and ion conduction (Fig. 2a–g, Supplementary Fig. 7a)^28^. Na_X_-QTT currents were non-inactivating and not modulated by voltage, and while Na_X_ has been proposed to function as a leak channel in vivo^13^, future studies are required to define the biophysical properties of wild-type channels.

In the absence of extracellular Ca^2+^ ions, Na_X_-QTT gives rise to a near-Ohmic leak current that does not discriminate between the tested monovalent cations (Fig. 3f). This finding challenges prior suggestions that Na_X_ is Na^+^-selective^18^ and confirms a key role for the DIII-lysine in Na_V_ channels^32^. Extracellular Ca^2+^ block of Na_X_-QTT produces outward-rectifying currents with only a small inward leak component at negative membrane potentials (Fig. 4a). Na_X_ may therefore function as a Na^+^-leak channel sensitive to modulation by extracellular Ca^2+^. Ca^2+^ block of Na_X_-QTT is ~10-fold more potent than a block on Na_V_ channels (Fig. 4a)^35^, but similar to the Ca^2+^ sensitivity of the distantly related NALCN Na^+^-leak channel^33^, perhaps suggesting a shared mechanism to avoid excessive depolarizing Na^+^ influx into cells.

Na_X_-QTT has pharmacological sensitivities expected for a Na_V_-like channel, including a block by TTX, lidocaine, quinidine, and loperamide (Fig. 4b, c)^1^. However, flecainide, ranolazine, or phenytoin did not produce strong inhibition (Fig. 4c), possibly reflecting unique central cavity-lining residues in Na_X_ (Supplementary Fig. 5g) or the absence of an inactivated state.

Lipids occlude the ion conduction pathway in our Na_X_ channel structures (Fig. 2c, d, Supplementary Fig. 6h). The phospholipid which plugs the S6-gate is reminiscent of cholesterol-like molecules bound in Na_V_ channels^22,23^, where the occupancy of these hydrophobic ligands appears to correlate with the up-conformation of a conserved DIV-S6-tyrosine side-chain implicated in drug block (Supplementary Fig. 6h, 10d)^24,26^. Drug binding can enforce a down-conformation of this S6-tyrosine, concurrent with the displacement of the lipid-like density at the S6-gate (Supplementary Fig. 10d)^26^. Thus, our Na_X_ structures advance an emerging paradigm in channels whereby membrane lipids can directly modulate pore structure, drug binding, and ion conductance.

Under the conditions that we have examined, the human Na_X_ channel does not function independently as a Na^+^-activated channel, nor as a voltage-activated channel. Using structure-guided engineering, we discovered a Na_X_-QTT channel construct that displays a non-selective cationic conductance and potent inhibition by extracellular Ca^2+^ and TTX. Overall, although the steps, factors, or pathways required for endogenous channel activation still need to be precisely defined, our study reveals that the Na_X_ channel may operate as a Ca^2+^-modulated Na^+^ leak channel and provides new tools to further demystify this once recalcitrant channel.

## Methods

### Protein expression and purification

Full-length human Na_X_ containing tandem 2xStrepII and 2xFLAG tags at its N-terminus and untagged full-length human β3 were each cloned into a pRK vector behind a CMV promotor. Constructs were co-transfected with PEI into Expi293 cells and cultured for 48 h in SMM 293T-I medium under 5% CO_2_ at 37 °C. Cells were harvested by centrifugation at 800 × *g* for 10 min and resuspended and lysed in Buffer A (25 mM ADA pH 6.0, 200 mM NaCl, 1 mM PMSF, 1 μg/mL benzonase, and 1× Roche protease inhibitor cocktail) by dounce homogenization. To solubilize the cell membranes, glyco-diosgenin (GDN) and cholesterol hemisuccinate (CHS) were added to the sample to final concentrations of 2% and 0.3%, respectively, and the sample was gently stirred at 4 °C for two hours. Insoluble material was then collected by ultracentrifugation at 125,000 × *g* for 1 h at 4 °C. Supernatants were applied to anti-FLAG M2 agarose resin that had been pre-equilibrated in Buffer B (25 mM ADA pH 6.0, 200 mM NaCl, 0.01% GDN) and bound in batch at 4 °C for 1 h. The sample was then applied to a gravity column and the collected resin was washed with 10 CV Buffer B followed by 10 CV supplemented with 5 mM ATP and 10 mM MgCl_2_. Protein was eluted with 5 CV Buffer B supplemented with 300 μg/mL FLAG peptide. Eluates were applied directly to Strep-Tactin XT Superflow high capacity resin that had been pre-equilibrated in Buffer B and bound in batch for three hours, then washed with 10 CV Buffer B prior to sample elution in 5 CV supplemented with 50 mM biotin. For nanodisc incorporation, the sample was concentrated to 15 μM using an Amicon Ultra centrifugal filter device (100 kDa MWCO). For structural analysis in detergent (GDN), the eluate was instead concentrated to 100 μL and applied to a Superose 6 3.2/300 column that had been pre-equilibrated in Buffer B.

### Mass spectrometry analysis

Following size exclusion chromatography, 10 µg of the Na_X_-containing peak fraction (co-expression with the β3-subunit) was denatured with 8 M guanidine (1:1 v/v), reduced with 1 M dithiothreitol (DTT, Sigma-Aldrich, St Louis, MO) to a final concentration of 100 mM DTT and incubated at 95 °C for 10 min, then centrifuged. 10 µL of 1 M Tris, pH 8 was added to the solution, and water was used to dilute the guanidine to a 2 M final concentration. Deglycosylation was performed with 2 µL of PNGaseF (15000 U, New England Biolabs) followed by overnight incubation at 37 °C. Samples were further reduced with 10 mM DTT at 60 °C (15 min) followed by alkylation with 20 mM iodoacetamide at room temperature. Proteins were digested with 0.2 µg trypsin (Promega) or chymotrypsin in 50 mM ammonium bicarbonate, pH 8 at 37 °C overnight. Digestions were quenched with formic acid and the supernatants were subjected to desalting on C18 PhyTips (PhyNexus), lyophilized, reconstituted in 0.1% formic acid containing 2% acetonitrile and analyzed without further processing by reversed-phase nano-LC/MS/MS on a Waters NanoAcquity HPLC system (Waters Corp.) interfaced to an Elite Orbitrap mass spectrometer (ThermoFisher Scientific). Peptides were loaded onto a Symmetry C18 column (1.7 mm BEH-130, 0.1 × 100 mm, Waters) and separated with a 60 min gradient from 2 to 25% solvent B (0.1% formic acid, 98% acetonitrile) at 1 µL/min flow rate. Peptides were eluted directly into the mass spectrometer with a spray voltage of 1.2 kV. Full MS data were acquired in FT for 350–1250 *m/z* with a 60,000-resolution. The most abundant ions from full MS scans were selected for MS/MS through a 2-Da isolation window.

Acquired tandem MS spectra were searched using the Mascot algorithm (Matrix Sciences) with trypsin or chymotrypsin enzyme specificity. Search criteria included a full MS tolerance of 50 ppm, MS/MS tolerance of 0.5 Da with oxidation of methionine (+15.9949 Da) as variable modification, and carbamidomethylation (+57.0215 Da) of cysteine as a static modification. Data were searched against the specific sequences of in-house constructs overlaid onto the Uniprot mammalian database, including reverse protein sequences. Peptide assignments were first filtered to a 2% false discovery rate (FDR) at the peptide level and subsequently to a 2% FDR at the protein level.

### Reconstitution of β3-Na_X_ into lipid nanodiscs

For reconstitution into nanodiscs, multiple 50 μL sample aliquots were each applied to 2 mL tubes with a 200-fold molar excess of a POPC:POPE:POPG lipid mix (3:1:1 ratio solubilized in a sonication bath at 10 mg/mL in a buffer containing 50 mM HEPES pH 7.5, 100 mM NaCl, 5 mM MgCl_2_, and 1% CHAPS) and incubated for 30 min at 4 °C. The membrane scaffold protein MSP1E3D1 (Sigma) was then applied to the samples in a 4-fold molar excess and incubated for an additional 30 min at 4 °C. Samples were then diluted to 1.5 mL and Bio-Beads were applied to a final concentration of 0.25 mg/mL. Samples were then nutated overnight at 4 °C prior to Bio-Bead removal. The samples were then pooled and passed over 1 mL Strep-Tactin XT resin in a column that had been pre-equilibrated in Buffer C (25 mM HEPES pH 7.0, 200 mM NaCl) to remove empty nanodiscs. The column was washed with 5 CV Buffer C prior to sample elution with 5 CV containing 50 mM biotin. The sample was then concentrated to 100 μL and applied to a Superose 6 3.2/300 column that had been pre-equilibrated in Buffer C. Peak fractions were pooled and concentrated to 3 mg/mL.

### Cryo-EM sample preparation and data acquisition

Samples were cross-linked with a final concentration of 0.05% EM-grade glutaraldehyde at room temperature for 10 min. The crosslinking reactions were quenched by the addition of 1 M Tris pH 7.0. Ultrafoil R2/2 (200 mesh) cryo-EM grids (Quantifoil GMBH) were plasma cleaned for 5 s using the Solarus plasma cleaner (Gatan, Pleasanton, CA). Three microliters of the final samples at 2.25 mg/mL (nanodiscs) or 1.5 mg/mL (GDN) were applied to the cryo-EM grids and blotted for 2.5 s using a Vitribot Mark IV (ThermoFisher Scientific, Waltham, MA) using a blot force setting of 8 at 100% humidity, and plunged into liquid ethane. Grids were then imaged on a Titan Krios electron microscope (ThermoFisher Scientific, Waltham, MA) operated at 300 keV with a bioquantum energy filter using a K2 (nanodiscs) or K3 (GDN) Summit direct electron detector (Gatan, Pleasanton, CA). Images of the nanodisc were recorded at a magnification of ×165,000, which corresponded to 0.824 Å/pixel using a 20 eV energy slit. Image stacks contained 50 images recorded at 0.2 s intervals over 10 s, giving a total exposure of ~50 e^−^/Å^2^. Images of the GDN sample were recorded in super-resolution mode at ×105,000 magnification, corresponding to 0.419 Å/pixel, using a 20 eV energy slit. Image stacks contained 60 images recorded at 0.05 s intervals over 3 s, giving a total exposure of ~64 e^−^/Å^2^. All data collection was done using serialEM^36^.

### Cryo-EM data processing

For the nanodisc sample, Cryo-EM data was processed using a combination of the WARP, RELION, and cisTEM software packages^37–40^. For the first dataset, 11,658 movies were corrected for frame movement using MotionCor2^41^ in RELION. The resulting images were filtered to retain only those with an accumulated motion total below a value of 250 and contrast-transfer function (CTF) parameters were fit using the 30–4.5 Å band of the spectrum using CTFFIND4.1^42^. Images were filtered to include only those with a detected fit resolution better than 5 Å, giving a total of 9988 good images for further processing. 1,669,107 particles were picked using a deep learning-based algorithm in WARP^37^. Particles were subjected to two rounds of 2D classification in cisTEM, and the best 30 classes were chosen (76,392 particles) and exported to RELION for 3D classification. A 20 Å low-pass filtered (LPF) K_V_1.2 reconstruction solved in nanodiscs (EMD-9024)^43^ for which all density outside of the nanodisc had been erased was used as the initial 3D reference. The best obtained 3D volume was then used as the reference for the second round of 3D classification using a broader selection of particles from the 2D classification in cisTEM (350,901 particles). The best 3D volume and its corresponding 91,435 particles were then imported back into cisTEM for iterative rounds of auto-refine without a mask and manual refinement with iteratively adjusted masks and 20 Å LPF outside the mask (outside weight of 0.8). The resulting 4.5 Å map was then used as the reference in a final round of unmasked auto-refinements and masked manual refinements in cisTEM using the particle stack from only a single round of 2D classification in cisTEM, giving the final 3.2 Å map.

For the GDN detergent sample, Cryo-EM data were processed using a combination of the RELION, Gautomatch, and cisTEM software packages^38–40,44^. 10,850 movies were corrected for frame movement using MotionCor2^41^ in RELION and binned to 1 Å/pixel. The resulting images were filtered to retain only those with an accumulated motion total below a value of 250 and contrast-transfer function (CTF) parameters were fit using the 30–4.5 Å band of the spectrum using CTFFIND4.1^42^. Images were filtered to include only those with a detected fit resolution better than 5 Å, giving a total of 10,569 good images for further processing. A total of 2,780,663 particles were automatically picked using Gautomatch^44^ using 16 uniform projections of the reconstruction of Na_V_1.4 in complex with the β1 auxiliary subunit (EMD-9617) as templates. Particles were subjected to a single round of 2D classification into 250 classes in RELION and a particle stack containing 1,420,422 particles from the best 55 classes were extracted and exported to cisTEM. The structure of β3-Na_X_ solved in nanodiscs was used as an initial reference in an initial round of auto-refine without a mask, followed by several rounds of manual refinement with iterative mask refinement and 20 Å LPF outside the mask (outside weight of 0.8). Manual refinement runs included defocus refinement and used a score threshold of 0.2. Iterative rounds of unmasked auto-refinements and masked manual refinements yielded a final reconstruction of 2.85 Å resolution.

### Model building and structure analysis

The structure of Na_V_1.4 in complex with the β1 auxiliary subunit (PDB 6AGF)^22^ was used as a template to generate a Na_X_ homology model using the Phyre2 server^45^. This model along with β1 was rigid body docked into the cryo-EM map for manual rebuilding in Coot^46^ to give an initial model of the β3-Na_X_ complex. Multiple rounds of real-space refinement in Phenix^47^ and manual rebuilding in Coot were followed by molecular dynamics-assisted manual refinement in UCSF ChimeraX^48^ with ISOLDE^49^. After a final refinement in Phenix, the model was validated using the Phenix validation package. Structure figures were generated using PyMol^50^, UCSF Chimera^51^, and UCSF ChimeraX. Caver3.0 was used to analyze the channel pore^52^. Sequence alignments were performed using Clustal Omega^53^ and rendered with ESPript 3.0^54^.

### Molecular biology for biochemical and electrophysiological experiments

Complementary DNAs (cDNAs) of human Na_X_, Na_X_-eGFP-2 × FLAG, Na_V_1.7, Na_X_-Na_V_1.7 domain-swapped chimeras, ATP1A1, ATP1B1, and SAP97, codon optimized for *Homo sapiens*, were cloned into the pcDNA3.1/Hygro^(+)^ vector. The human Na_V_β1, Na_V_β3, Ca_V_β1, Ca_V_γ2, Ca_V_α2δ1, ATP1A1, ATP1B1, ATP1G1, and SAP97 constructs were cloned into the pcDNA3.1^(+)^ vector. For expression in *Xenopus laevis* oocytes, cDNAs were linearized using either NotI, BamHI, or XbaI restriction enzyme and then transcribed to capped RNAs with the T7 mMessage mMachine Kit (Ambion). For expression in HEK293T and Neuro-2a cells, plasmid DNAs purified with the NucleoBond Xtra Midi Plus kit (Macherey-Nagel) were used.

### Two-electrode voltage-clamp electrophysiology

Oocytes were prepared as previously described^33^. Stage V/VI oocytes were obtained from ovaries of female *Xenopus laevis* frogs (obtained from African Reptile Park, South Africa). Animals were anaesthetized in 0.3% tricaine. Animal work and handling were carried out under license 2014–15-0201–00031, approved by the Danish Veterinary and Food Administration. Frogs were housed and cared for by an animal facility with ethical approval from the University of Copenhagen, Denmark. Healthy-looking stage V–VI oocytes were injected with 2.5–40 ng of RNA (in 5–41 nL) using a Nanoliter 2010 injector (World Precision Instruments). When excluding one or more constructs, an equivalent volume of nuclease-free water was added to maintain a constant RNA concentration across different construct combinations. Injected oocytes were kept at 18 °C, 140 rpm, in ND96 supplemented with 50 µg/mL gentamicin and 50 µg/mL tetracycline (in mM: 96 NaCl, 2 KCl, 1 MgCl_2_, 1.8 CaCl_2_, 2.5 sodium pyruvate, 0.5 theophylline, 5 HEPES; pH 7.4 with NaOH) for 2–5 days. Two-electrode voltage-clamp measurements were performed at room temperature using a Warner OC-725C Oocyte Clamp amplifier (Warner Instrument Corp, USA) and under constant perfusion with ND96 solution (in mM: 96 NaCl, 2 KCl, 1 MgCl_2_, 1.8 CaCl_2_, 5 HEPES; pH 7.4 with NaOH). For ion-selectivity experiments, external solutions contained 115 mM of test cations as chloride salts, 1.2 mM CaCl_2_, 2 mM MgCl_2_, 5 mM HEPES (pH 7.4 with the corresponding hydroxide). Data acquisition was performed using a Digidata 1550 digitizer (Molecular devices; sampled at 10 kHz) and pCLAMP 10 software (Molecular Devices). Microelectrodes from borosilicate glass capillaries (Harvard Apparatus) were prepared to have resistances around 0.2–1.0 MΩ using a P-1000 Flaming/Brown Micropipette Puller System (Sutter Instrument) and backfilled with 3 M KCl. The concentrations of compounds shown in Fig. 1b are as follows: aconitine (300 µM); veratridine (100 µM); blood depressing substance I (BDS-I, 300 nM); and neurotoxin 2 (ATX-II, 100 nM). Concentrations of compounds shown in Fig. 4c (middle panel) are as follows: tetrodotoxin (TTX, 10 µM); lidocaine hydrochloride (10 mM); quinidine (100 µM); loperamide hydrochloride (100 µM); flecainide acetate (300 µM); ranolazine dihydrochloride (300 µM); phenytoin sodium (300 µM); zinc chloride (ZnCl_2_; 1 mM); cobalt chloride (CoCl_2_; 1 mM); and gadolinium chloride (GdCl_3_; 100 µM).

### Cell culture, cell surface biotinylation, and western blots

HEK293T and Neuro-2a cells (ATCC) were grown and maintained as described previously^33^. Approximately 800,000 HEK293T and 250,000 Neuro-2a cells were seeded in 35 mm cell culture dishes ~20 h before transient transfection with 1 µg of cDNA using LipoD293 ver. II (tebu-bio). For biochemical experiments, the cDNAs of Na_X_-eGFP-2 × FLAG and β3 were mixed in a mass ratio of 1:1. Equal amount of empty vector DNA was added to keep the total cDNA amount constant when β3 was excluded from transfection. Transfected HEK293T cells were used for biochemical experiments 24 h post-transfection. To induce differentiation, Neuro-2a cells were serum-starved 24 h post-transfection (cultured in DMEM for an additional 24–30 h) before they were used for biochemical and electrophysiological experiments. Cell surface biotinylation and western blots were performed as described previously^33^, with only slight modifications: (1) The quenching step was performed under gentle agitation on ice for 30 min, (2) *Xenopus laevis* oocytes were washed twice with tris-buffered saline before lysis, and (3) the total lysate fraction for oocytes was diluted 1:5 in SDS sample buffer before loading onto the gel due to excess protein. Antibodies were used as stated in ref. ^33^, except the mouse anti-Na^+^/K^+^-ATPase antibody was from Santa Cruz Biotechnology (sc-21712). Blots are representative of a minimum of three individual experiments.

### Patch-clamp electrophysiology

Differentiated Neuro-2a cells expressing Na_X_-eGFP-2 × FLAG were seeded on glass coverslips an hour before patch-clamping experiments. Cells were voltage-clamped at −60 mV in whole-cell configuration (Fig. 1e–g; Supplementary Fig. 1f) using an Axopatch 200B amplifier (Molecular Devices). Digidata 1550 A digitizer (Molecular devices; sampled at 10 kHz) and pCLAMP 10 software (Molecular Devices). Glass pipettes for patch-clamp experiments were pulled from borosilicate glass capillaries (World Precision Instruments; Kwik-Fil 1.5/1.12; OD/ID), and fire polished to final resistances between 2.0 and 5.5 MΩ. Extracellular solutions contained (in mM): 140 or 190 NaCl, 5 KCl, 2.5 CaCl_2_, 1 MgCl_2_, 5 HEPES, 20 glucose (pH 7.4 with NaOH). The osmolarity values of these solutions were ~302 mOsm/L (for 140 NaCl) and ~395 mOsm/L (for 190 NaCl). The intracellular solution contained (in mM): 120 K-gluconate, 20 TEA-Cl, 2 MgCl_2_, 2Na_2_ATP, 1 EGTA and 10 HEPES, pH 7.3 with KOH (~281 mOsm/L).

HEK293T cells expressing Na_X_- or Na_X_-QTT-eGFP-2 × FLAG were seeded on glass coverslips an hour before whole-cell patch-clamping experiments. For a more physiological condition (Fig. 3d; Supplementary Fig. 1b), the extracellular solution contained 150 mM NaCl, 5 mM KCl, 0.5 mM CaCl_2_, 1.2 mM MgCl_2_, 10 mM HEPES, and 13 mM D-(+)-glucose (pH 7.4) with NaOH, ~320 mOsm/L, and the intracellular solution contained 140 mM CsCl, 10 mM CsF, 5 mM EGTA, 10 mM HEPES, and 2 mM Na_2_ATP (pH 7.2) with CsOH, ~304 mOsm/L. To determine if Na_X_-QTT was cation-permeable, we first substituted all extracellular cations with NMDG^+^ (Fig. 3e). Extracellular solution contained 150 mM NMDG and 10 mM HEPES (D-(+)-glucose was added accordingly to achieve osmolarity ~325 mOsm/L and pH was adjusted to 7.4 with hydrochloric acid). To determine if Na_X_-QTT was anion-permeable, we substituted all Cl^−^ ions in both intra- and extracellular solutions with the large anion methanesulfonate (MS^−^) (Supplementary Fig. 9d). The extracellular solution contained 150 mM NaMS and 10 mM HEPES (D-(+)-glucose was added accordingly to achieve osmolarity ~325 mOsm/L, and pH was adjusted to 7.4 with NaOH). The intracellular solution contained 136 mM NaMS, 10 mM NaF, 5 mM EGTA, 10 mM HEPES, and 2 mM Na_2_ATP (pH 7.2 with NaOH, ~309 mOsm/L). The extracellular NMDG solution contained 150 NMDG and 10 mM HEPES (D-(+)-glucose was added accordingly to achieve osmolarity ~325 mOsm/L, and pH was adjusted to 7.4 with methanesulfonic acid).

For ion-selectivity experiments (Figs. 3e, f, 4a) (i) involving monovalent cations, the extracellular solution contained 150 mM XCl, 10 mM HEPES, and D-(+)-glucose was added accordingly to achieve osmolarity ~325 mOsm/L, and the pH was adjusted to 7.4 with XOH, where X indicates the cation of interest (Na^+^, K^+^, Li^+^, or Cs^+^); (ii) involving Ca^2+^, the extracellular solution contained 110 mM CaCl_2_, 10 mM HEPES, and D-(+)-glucose was added accordingly to achieve osmolarity ~325 mOsm/L, and the pH was adjusted to 7.4 with Ca(OH)_2_; (iii) the intracellular solution contained 136 mM NaCl, 10 mM NaF, 5 mM EGTA, 10 mM HEPES, and 2 mM Na_2_ATP (pH 7.2) with NaOH, ~309 mOsm/L.

### Data analysis

For data analysis, raw current traces were typically filtered using 8-pole Bessel low-pass filter at 500–800 Hz. Current traces were subjected to factor 5 data reduction for display in figures. Data are presented as mean ± standard deviation (SD) from at least 3 cells from at least two batches of cells. For ion-selectivity experiments performed with Na^+^ intracellular solution (Figs. 3f, 4a), liquid junction potential (LJP) was measured with reference to the Na^+^ intracellular solution and corrected after recording. Relative ion permeabilities (P_Na_/P_X_) were calculated using the equation P_X_/P_Na_ = [Na^+^]_IC_ exp(E_rev_F/RT)/[X^+^]_EC_ (X = Na, K, Li, or Cs), where F is Faraday’s constant, R is the gas constant, T = 274 K, and E_rev_s were measured from the −80 to +80 mV ramp (corrected for LJP). Data are presented as mean ± s.d. from at least two batches of cells. Statistical comparisons were performed using GraphPad Prism (v.8.1, GraphPad Software) and the tests are mentioned in text.

### Reporting summary

Further information on research design is available in the Nature Research Reporting Summary linked to this article.

## Supplementary information

Supplementary Information

Reporting Summary

## Data Availability

The coordinates of the Na_X_-β3 structures deterimined in nanodiscs and GDN have been deposited in the Protein Data Bank under accession codes 7TJ8 and 7TJ9, respectively. Cryo-EM maps have been deposited in the Electron Microscopy Data Bank under accession codes EMD-25919 and EMD-25920. Source data are provided with this paper.

## Source data

Source Data
