# Supplementary material for: Structure-guided unlocking of NaX reveals a non-selective tetrodotoxin-sensitive cation channel
Source: Nat Commun. 2022 Mar 17;13:1416. doi: 10.1038/s41467-022-28984-4 (PMC8931054; doi:10.1038/s41467-022-28984-4)
Supplement: Supplementary file 1 — Supplementary Information [file 41467_2022_28984_MOESM1_ESM.pdf]

SUPPLEMENTARY INFORMATION FOR:

Structure-guided unlocking of Na_X reveals a non-selective tetrodotoxin-sensitive cation channel

Cameron L. Noland[1,#,&], Han Chow Chua[2,#], Marc Kschonsak[1,#], Stephanie Andrea Heusser[2], Nina Braun[2], Timothy Chang[3], Christine Tam[3], Jia Tang[4], Christopher P. Arthur[1], Claudio Ciferri[1,*], Stephan Alexander Pless[2,*], Jian Payandeh[1,*,%]

[1]Department of Structural Biology, Genentech Inc., South San Francisco, CA, USA 94080
[2]Department of Drug Design and Pharmacology, University of Copenhagen, Copenhagen, Denmark, 10 DK 2100
[3]Department of BioMolecular Resources, Genentech Inc., South San Francisco, CA, USA 94080
[4]Department of Microchemistry, Proteomics & Lipidomics, Genentech Inc., South San Francisco, CA, USA 94080

[#]These authors contributed equally: C.L.N., H.C.C., M.K.
[&]Current address (C.L.N): Department of Computational and Structural Chemistry, Merck Research Labs, South San Francisco, CA 94080
[%]Current address (J.P.): Department of Proteomics and Bioinformatics, Interline Therapeutics, South San Francisco, CA, USA, 94080
[*]Corresponding authors: ciferri.claudio@gene.com, stephan.pless@sund.ku.dk, jpayandeh@interlinetx.com

**Supplementary Figure 1.**	Evaluation of human Na_X in different expression systems.	page 2-3
**Supplementary Figure 2.**	Multiple-sequence alignment of Na_X and Na_V channels.	page 4-6
**Supplementary Figure 3.**	Na_X channel sample purification.	page 7
**Supplementary Figure 4.**	Cryo-EM workflow for Na_X-nanodisc sample.	page 8
**Supplementary Figure 5.**	Overall Na_X structure and comparison to Na_V channels.	page 9
**Supplementary Figure 6.**	Cryo-EM workflow for Na_X-GDN detergent sample.	page 10
**Supplementary Figure 7.**	Na_X DIII-DIV linker and Na_V1.7-Na_X chimeric channels.	page 11-12
**Supplementary Figure 8.**	Characterization of human Na_X carrying targeted pore-wetting S6-mutations.	page 13
**Supplementary Figure 9.**	Comparison of the Na_X and Na_V1.7 selectivity filters.	page 14
**Supplementary Figure 10.**	Human Na_X has atypical voltage sensor-like domains but a common hydrophobic S6 gate.	page 15
**Supplementary Table 1.**	Cryo-EM data collection, refinement and validation statistics	page 16

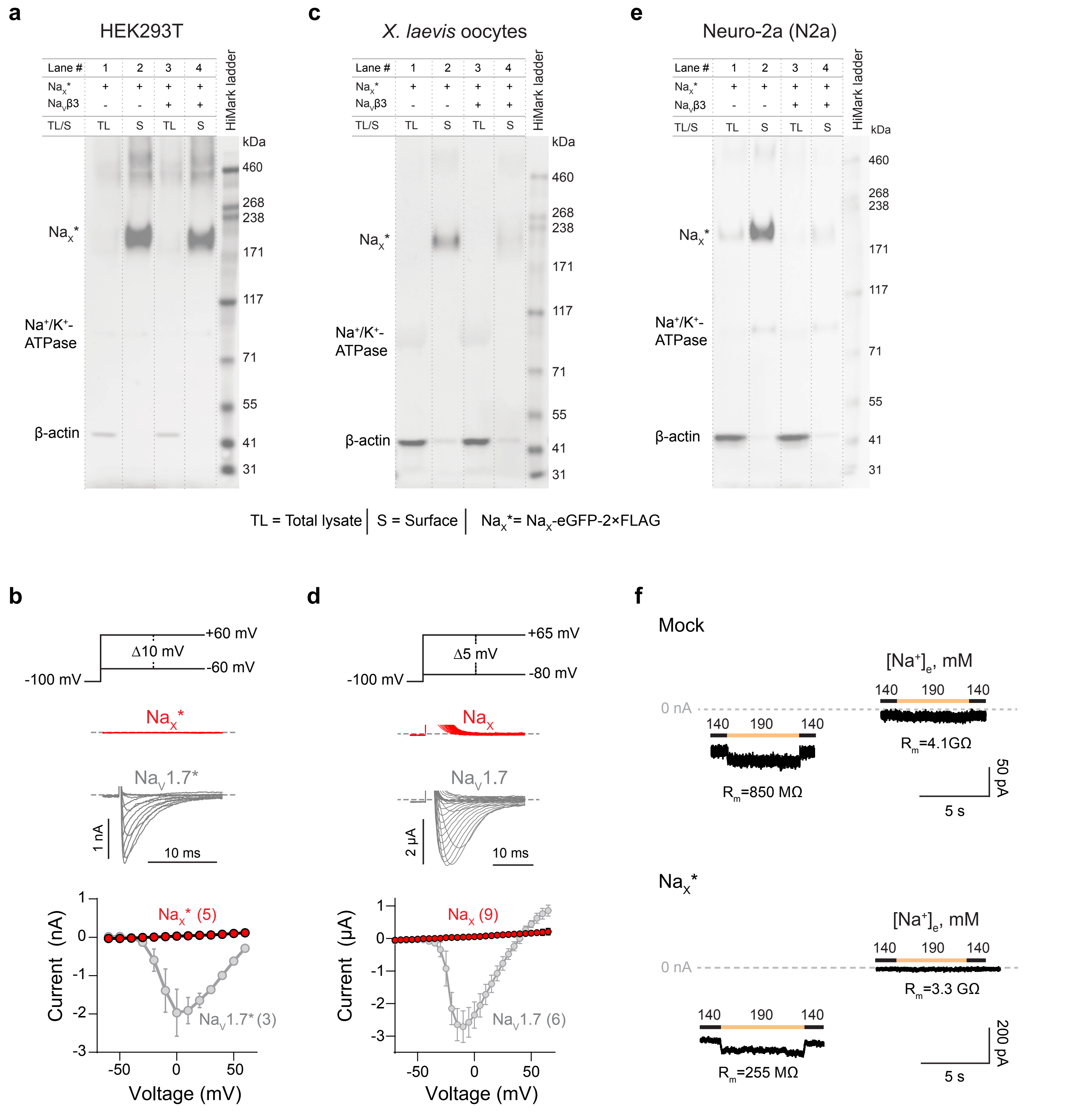

Supplementary Figure 1. Evaluation of human Na_x in different expression systems.

a. Western blots of total lysate and surface fraction of proteins extracted from HEK293T cells expressing the indicated constructs with C-terminal GFP and Flag tags on Na_x. Data represent three independent biological replicates.

b. Representative currents from HEK293T cells expressing human Na_x or $Na_V1.7$ (with C-terminal GFP and Flag tags) with indicated voltage protocol. Data are shown as mean ± SD. Numbers of biological replicates (*n*) are indicated.

c. Western blots of total lysate and surface fraction of proteins extracted from Xenopus laevis oocytes expressing the indicated constructs with C-terminal GFP and Flag tags on Na_x. Data represent three independent biological replicates.

d. Representative currents from oocytes expressing untagged human Na_x or NaV1.7 with indicated voltage protocol. Data are shown as mean ± SD. Numbers of biological replicates (n) are indicated.

e. Western blots of total lysate and surface fraction of proteins extracted from murine Neuro-2A cells expressing the indicated constructs with C-terminal GFP and Flag tags on Na_x. Data represent three independent biological replicates.

f. Representative currents from Neuro-2A cells expressing human Na_x (with C-terminal GFP and Flag tags) under indicated extracellular Na^+ concentrations (HP=-60 mV). Seal resistances (R_m) of individual cells are provided.

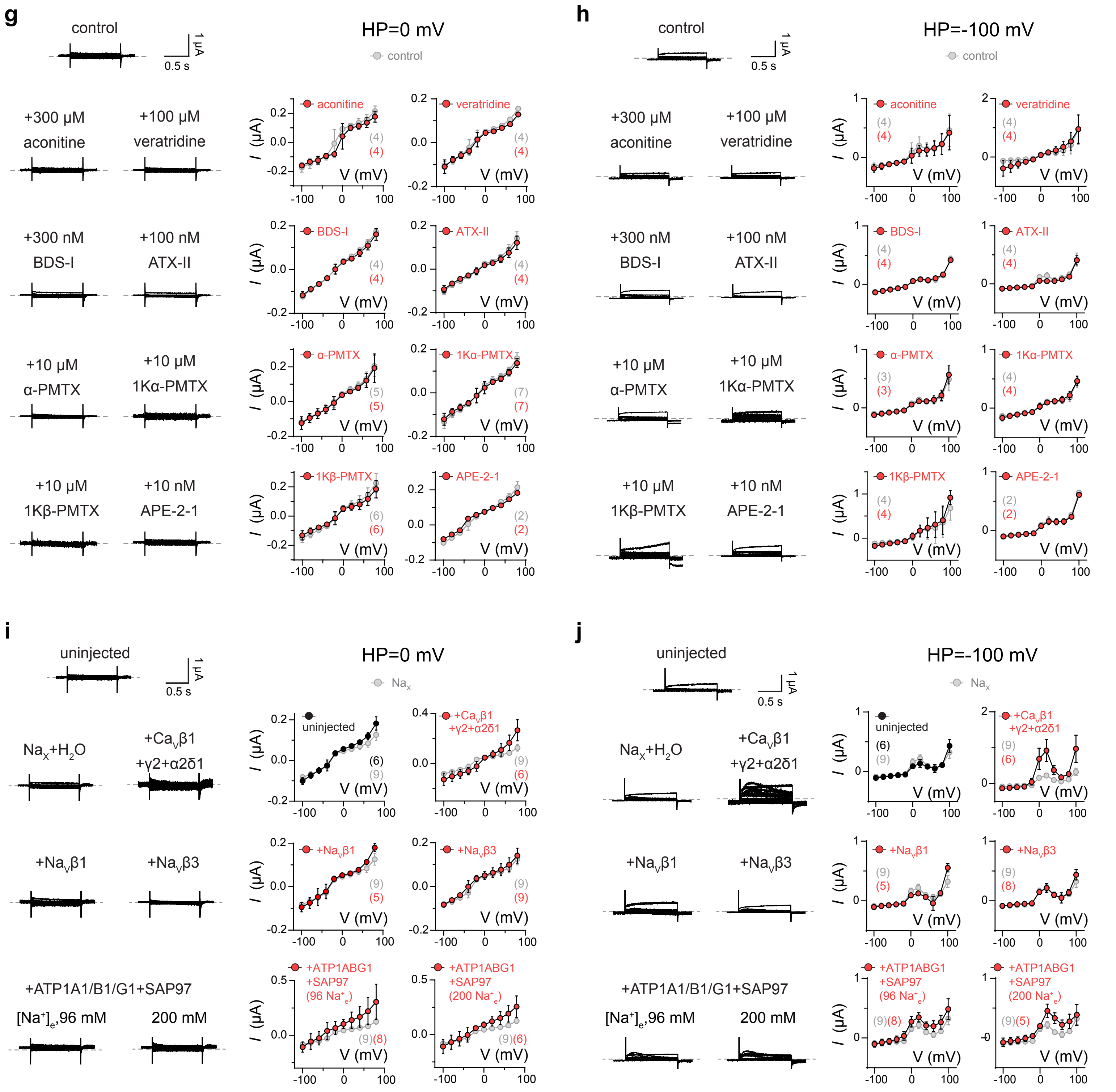

Supplementary Figure 1 (continued).

g-h. Representative currents from oocytes expressing Na_x in response to extracellular application of indicated compounds using two different voltage-step protocols (BDS-I: Blood depressing substance I; ATX-II: Neurotoxin 2; α-PMTX: α-Pompilidotoxin; 1Kα-PMTX: 1Kα-Pompilidotoxin; 1Kβ-PMTX: 1Kβ-Pompilidotoxin; APE-2-1: Anthopleurin-C). g, steps between +80 to -100 mV, in 20 mV increments, from a HP of 0 mV; h, steps between -100 to +100 mV, in 20 mV increments, from a HP of -100 mV. Right, shows I-V curve data summary from two independent experiments. Data are shown as mean ± SD. Numbers of biological replicates (*n*) are indicated.

i-j. Representative currents from oocytes co-expressing Na_x with Na_v and Ca_v auxiliary subunits, Na^+/K^+-ATPase α and β subunits, or synapse-associated protein 97 (SAP97) in response to two different voltage-step protocols. **i,** steps between +80 to -100 mV, in 20 mV increments, from a HP of 0 mV; **j,** steps between -100 to +100 mV, in 20 mV increments, from a HP of -100 mV. Right, shows I-V curve data summary from two independent experiments. Data are shown as mean ± SD. Numbers of biological replicates (*n*) are indicated.

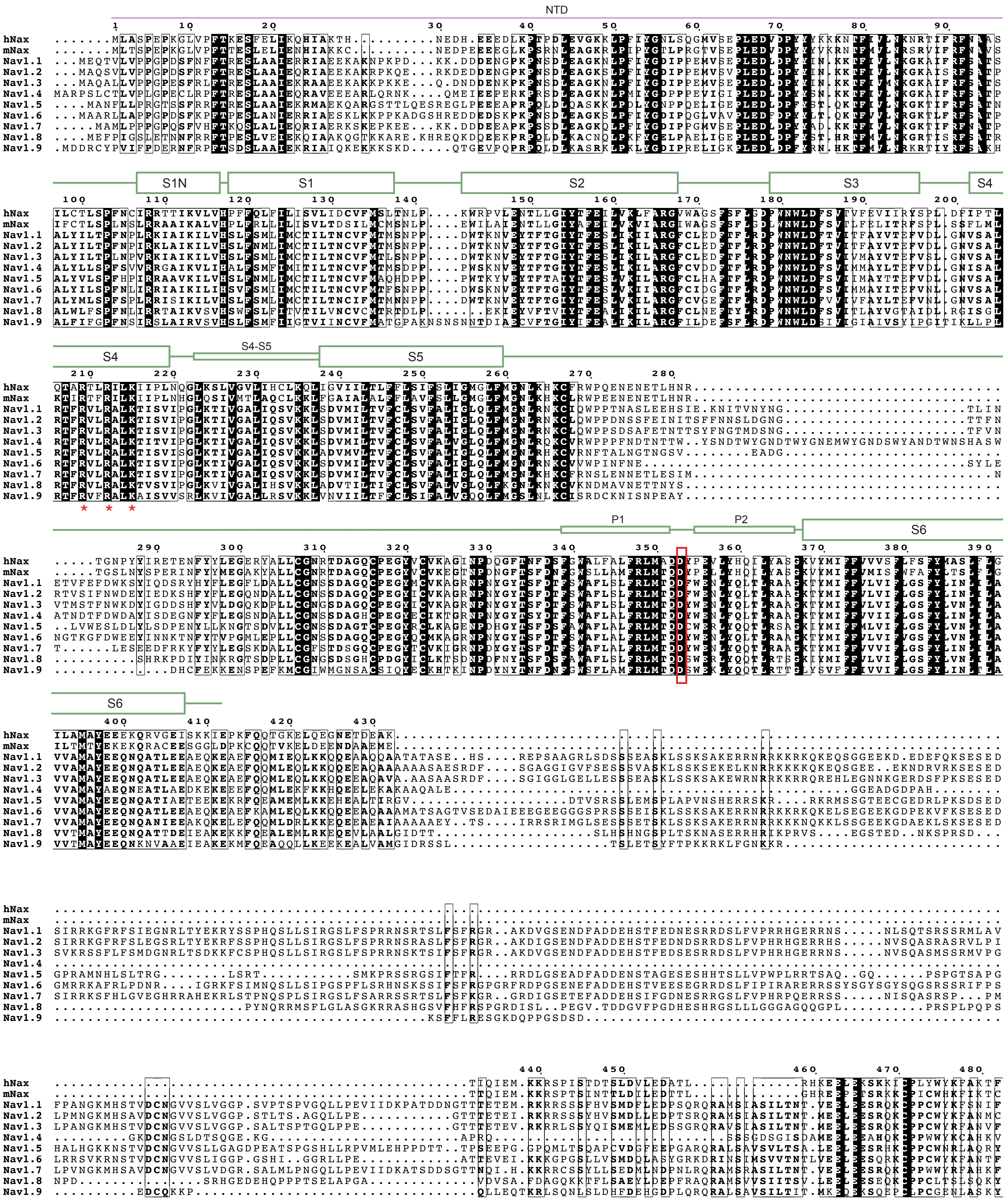
NTD
S1N
S1
S2
S3
S4
S4-S5
S5
P1
P2
S6
hNax
mNax
Nav1.1
Nav1.2
Nav1.3
Nav1.4
Nav1.5
Nav1.6
Nav1.7
Nav1.8
Nav1.9

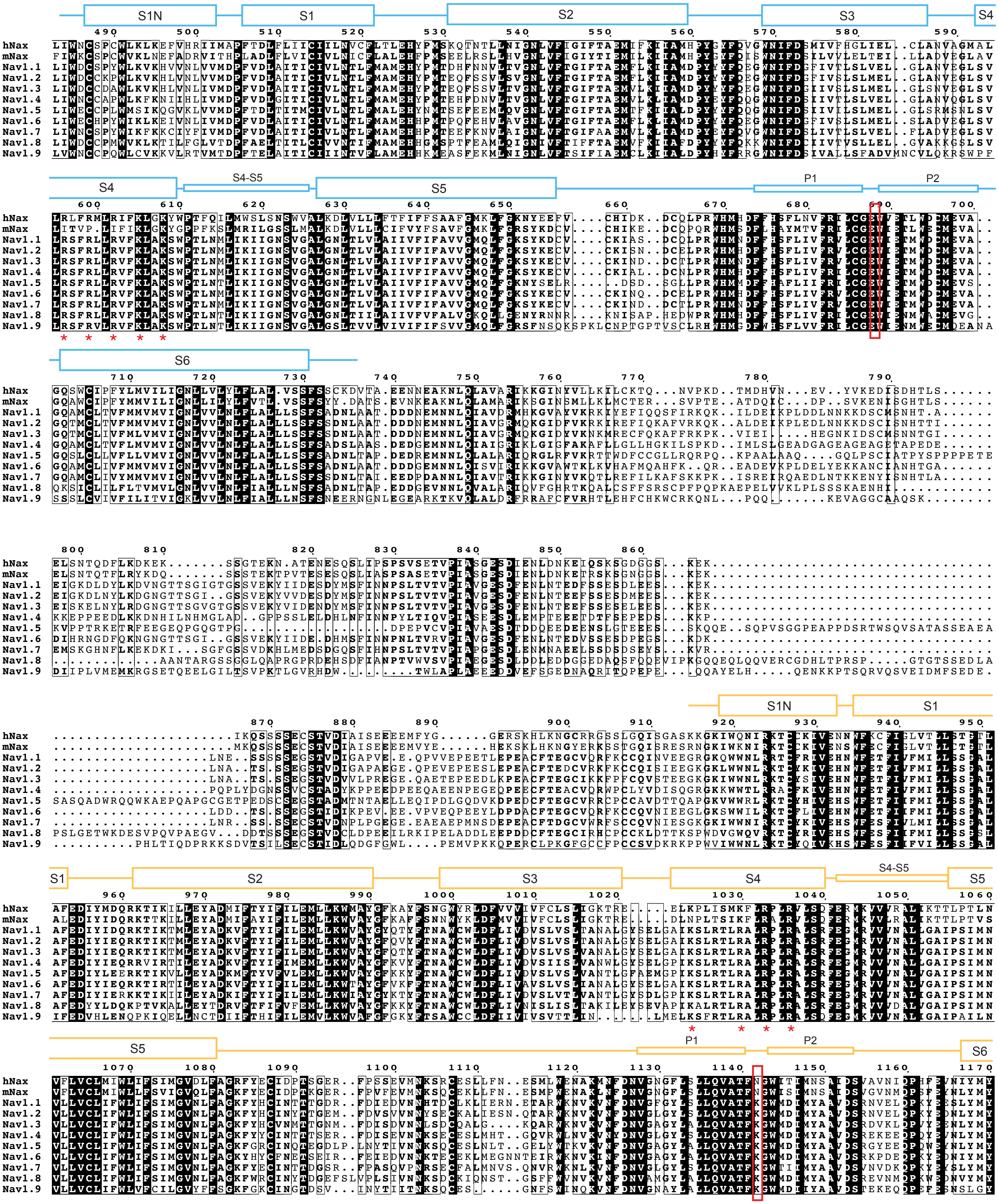

S1N
S1
S2
S3
S4
S4-S5
S5
P1
P2
S6
hNax
mNax
Nav1.1
Nav1.2
Nav1.3
Nav1.4
Nav1.5
Nav1.6
Nav1.7
Nav1.8
Nav1.9

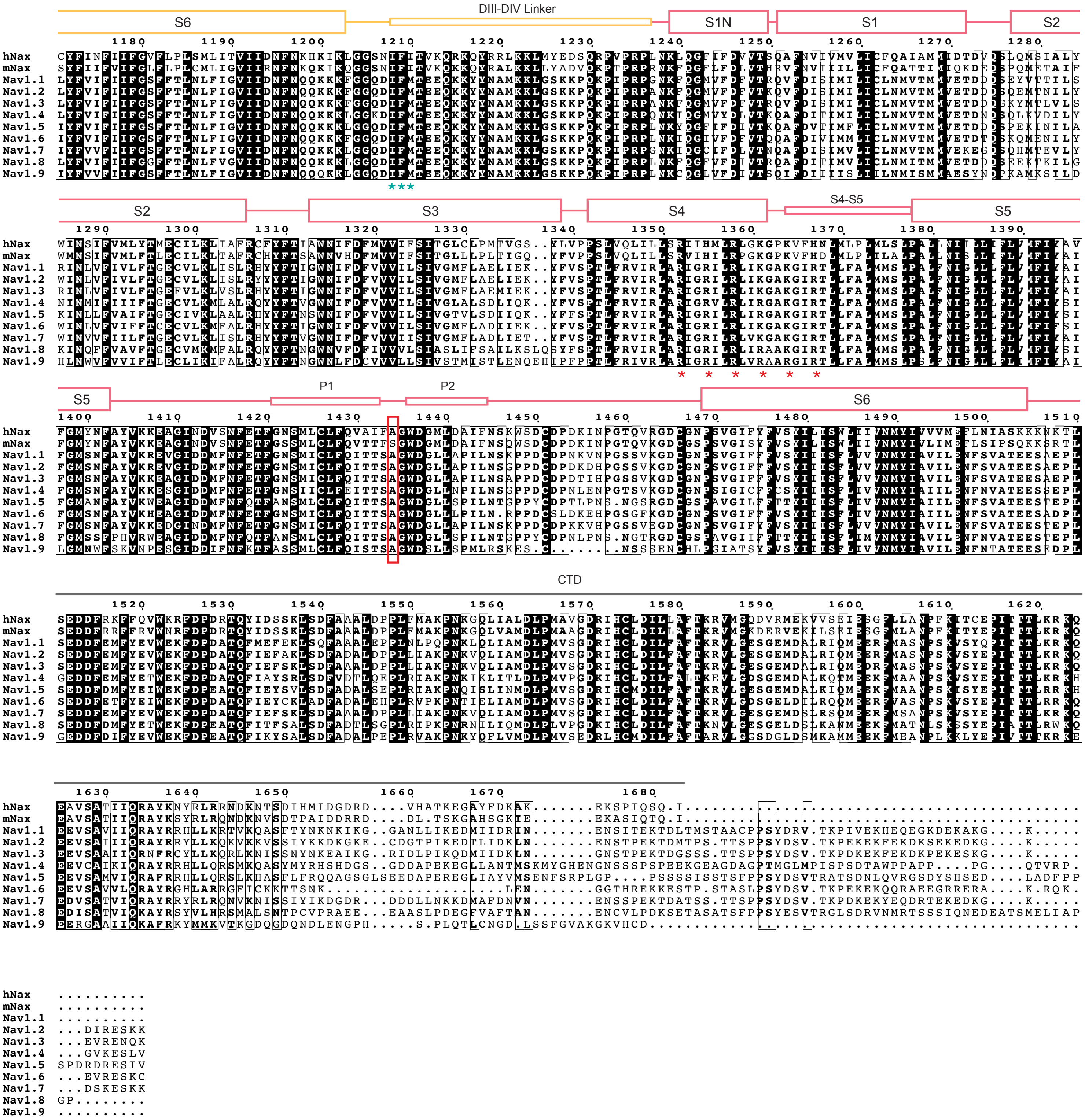

Supplementary Figure 2. Multiple-sequence alignment of Na_x and Na_v channels.
Human Na_x shares highest sequence identity with human $Na_v1.7$ (~55%) among human Na_v channels. Na_x domain boundaries are shown for reference. Na_x gating charges are denoted by red asterisks, selectivity filter residues are boxed in red, and the DIII-DIV linker IFI-motif is denoted by green asterisks.

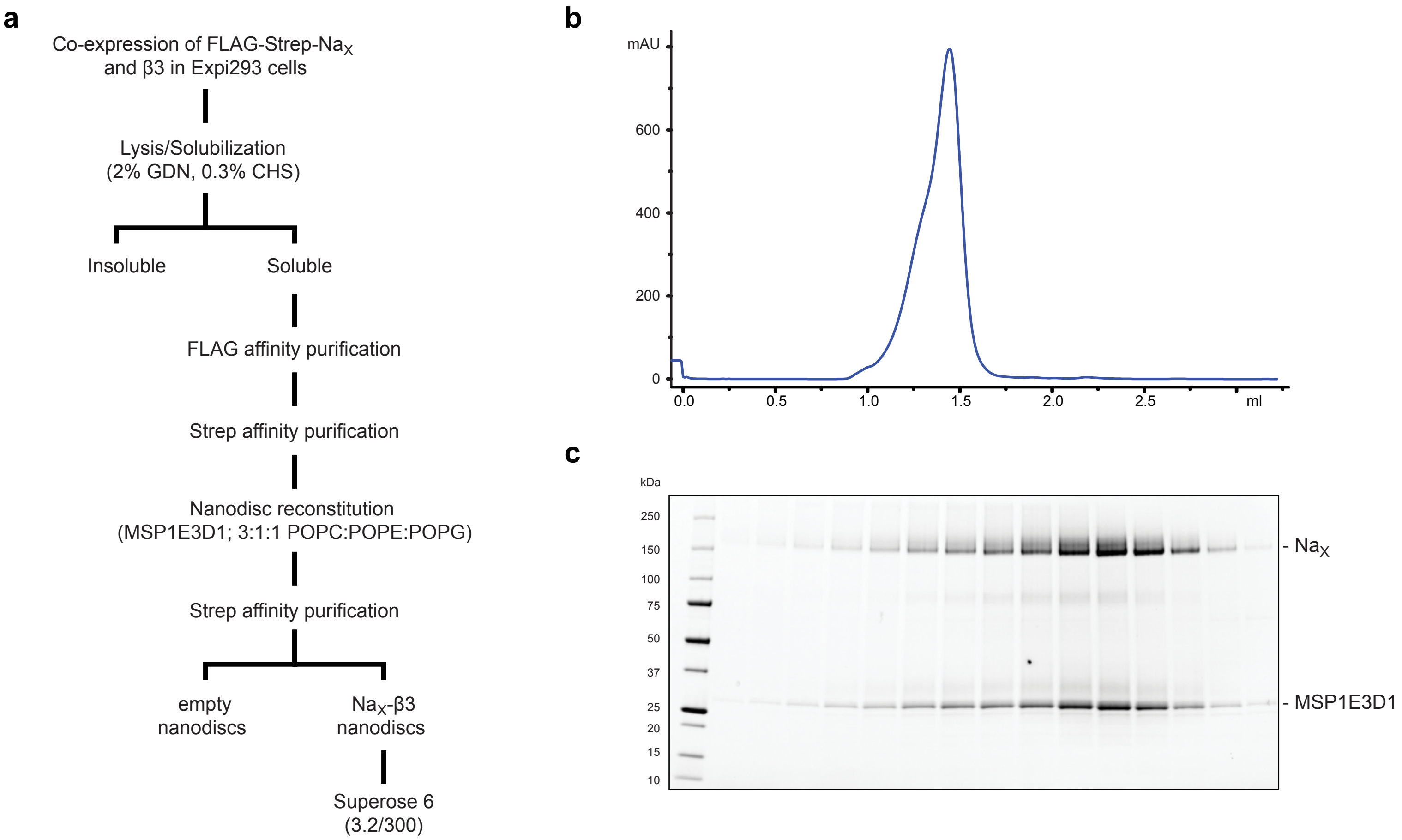

Supplementary Figure 3. Na$_x$ channel sample purification.
a. Na$_x$ expression, purification and reconstitution scheme.
b. Size-exclusion chromatography elution profile for β3-Na$_x$ sample in lipid nanodiscs (MSP1E3D1).
c. SDS-PAGE analysis of size exclusion chromatography elution fractions.

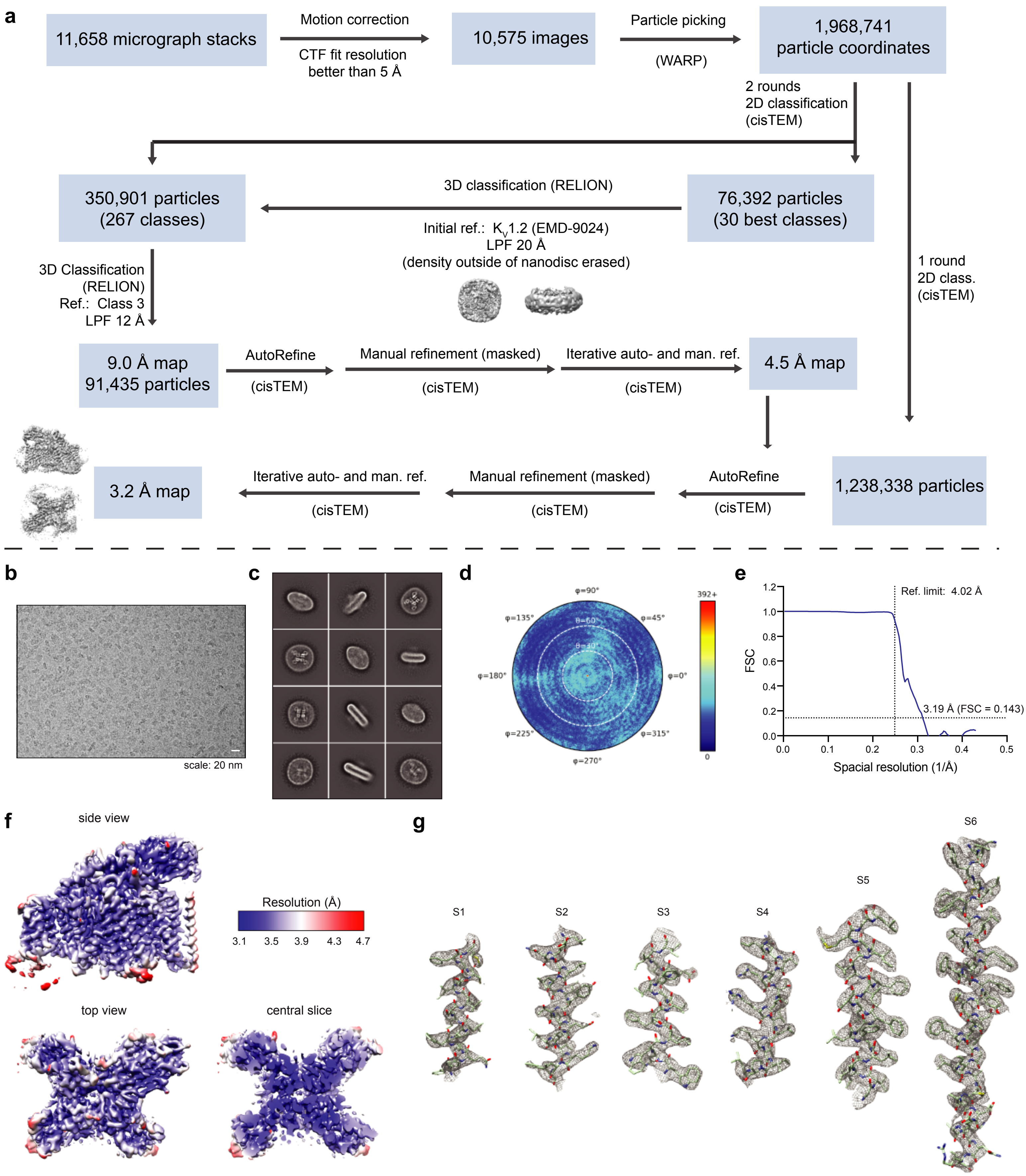

Supplementary Figure 4. Cryo-EM workflow for Na_X-nanodisc sample.
a. Schematic of the cryo-EM data processing workflow for the β3-Na_X-nanodisc sample **b.** Representative cryo-EM micrograph of β3-Na_X-nanodisc sample. **c.** Representative 2D class averages. **d.** Heat map showing the overall distribution of assigned particle orientations in the final reconstruction. **e.** Global resolution estimate based off the Fourier Shell Correlation (FSC) between two half datasets. **f.** Isosurface rendering of the final 3D reconstruction colored by local resolution, as estimated by windowed FSCs. **g.** Cryo-EM map shown over transmembrane regions of DI.

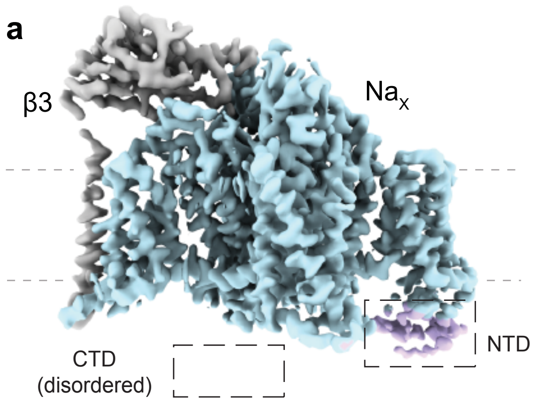

b

	hNa$_V$1.1 (PDB: 7DTD)	hNa$_V$1.2 (PDB: 6J8E)	hNa$_V$1.4 (PDB: 6AGF)	hNa$_V$1.5 (PDB: 6LQA)	hNa$_V$1.7 (PDB: 6J8H)	hNa$_x$-GDN
hNa$_x$-nanodisc	1.78 Å	1.80 Å	1.56 Å	1.78 Å	1.66 Å	0.96 Å
hNa$_x$-GDN	1.66 Å	1.60 Å	1.38 Å	1.69 Å	1.49 Å	

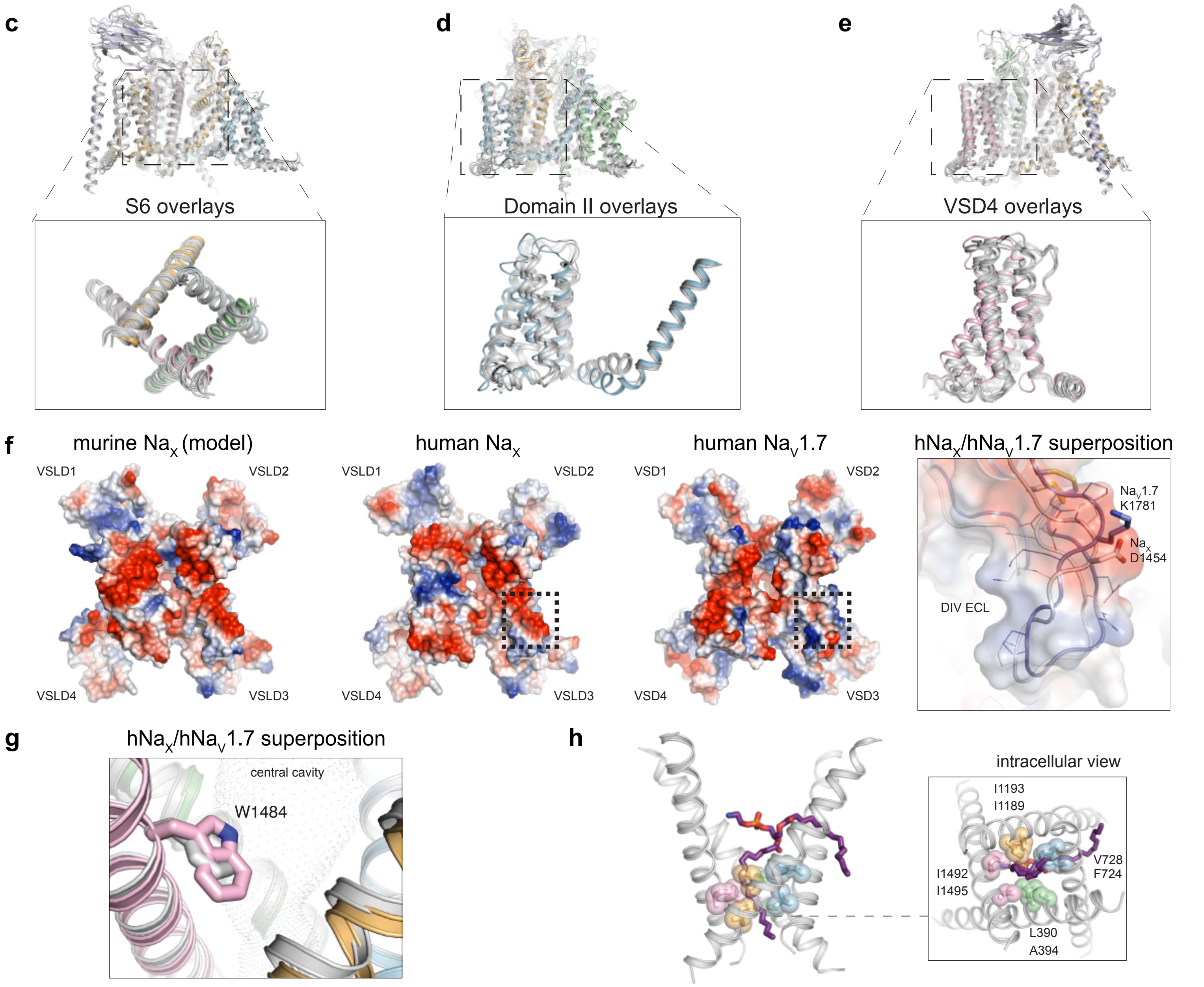

Supplementary Figure 5. Overall Na$_x$ structure and comparison to Na$_V$ channels.

a. Cryo-EM reconstruction of the β3-Na$_x$-nanodisc complex. Na$_x$ is colored blue, amino-terminal domain (NTD) is purple, and the β3-subunit grey. Approximate membrane boundaries are indicated. **b.** Overall root mean square deviation (RMSD) calculated between human Na$_x$ structures and indicated human Na$_V$ channel structures. **c-e.** Close-up views of β3-Na$_x$-nanodisc complex aligned with human Na$_V$1.2 (PDB 6J8E), human Na$_V$1.4 (PDB 6AGF), human Na$_V$1.5 (PDB 6UZ0) and human Na$_V$1.7 (PDB 6J8J) structures from various perspectives.
f. Extracellular view and electrostatic surface representations of, murine Na$_x$ (homology model), human Na$_x$ and human Na$_V$1.7 (PDB 6J8J). On right, a close-up view of an electronegative DIV extracellular loop (ECL) in Na$_x$ indicates a high local sequence and structural conservation with human Na$_V$1.7 (PDB 6J8J) except for a single residue difference (D1454 vs K1781). Additionally, no cryo-EM density for cations or identifiable cation binding sites are observed in this region. Note, β-subunits have been omitted for clarity. **g.** View into the central cavity highlighting the DIV W1484 side-chain of Na$_x$ with Na$_V$1.7 (PDB 6J8J) superimposed. DIV Phe of Na$_V$1.7 is shown in grey stick representation. **h.** Side-view of the β3-Na$_x$-nanodisc structure with S6 gate-lining side-chains and phosphatidylethanolamine shown in stick representation.

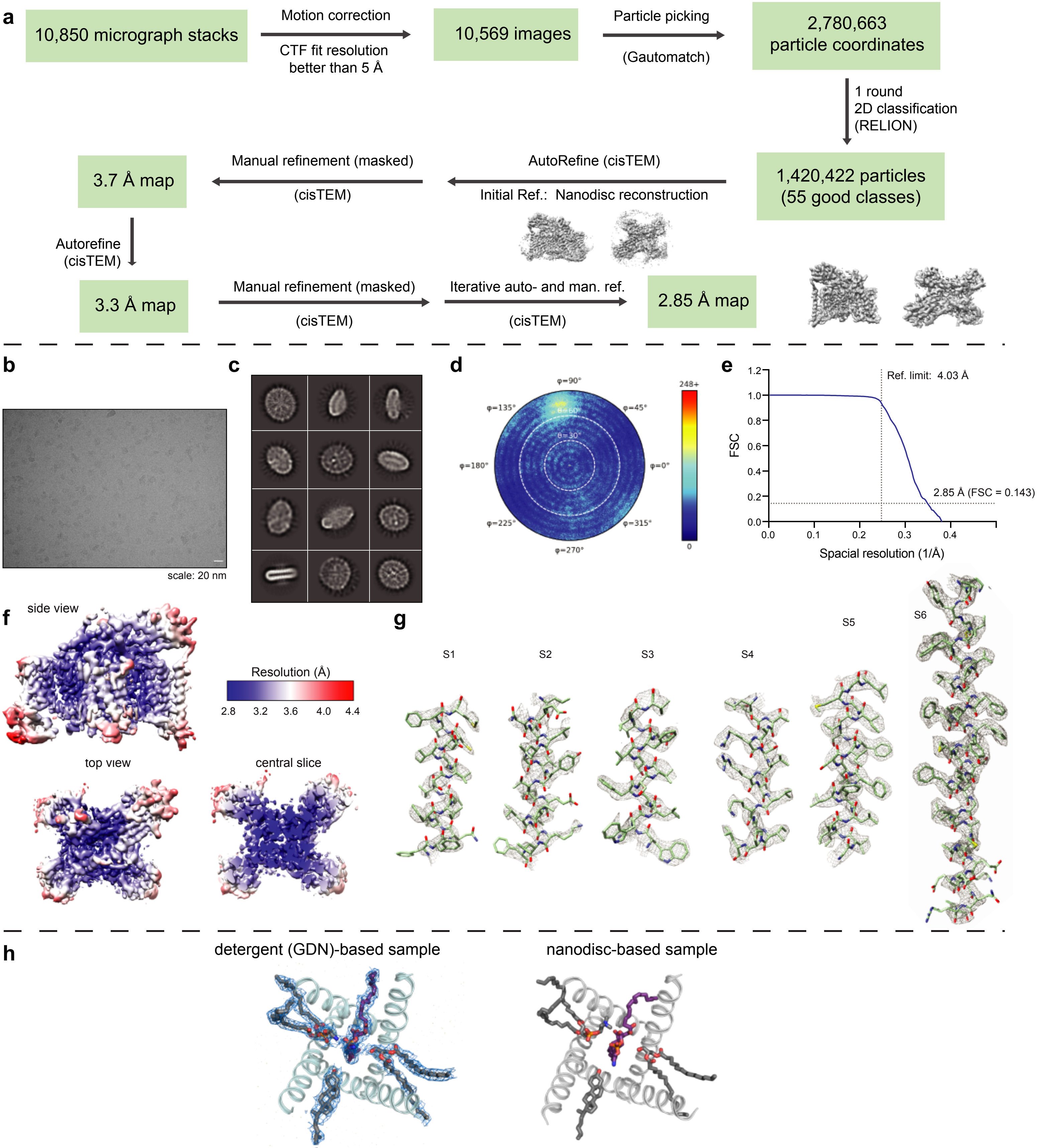

Supplementary Figure 6. Cryo-EM workflow for Na$_X$-GDN detergent sample.
a. Schematic of the cryo-EM data processing workflow for the β3-Na$_X$-detergent sample. **b.** Representative cryo-EM micrograph of β3-Na$_X$-detergent (GDN) sample. **c.** Representative 2D class averages. **d.** Heat map showing the overall distribution of assigned particle orientations in the final reconstruction. **e.** Global resolution estimate based off the Fourier Shell Correlation (FSC) between two half datasets. **f.** Isosurface rendering of the final 3D reconstruction colored by local resolution, as estimated by windowed FSCs. **g.** Cryo-EM map shown over transmembrane regions of DI. **h.** Views sliced through the pore module highlighting bound lipids with cryo-EM map for the GDN sample shown in blue mesh representation. The phosphatidylethanolamine that crosses the S6-gate is colored purple.

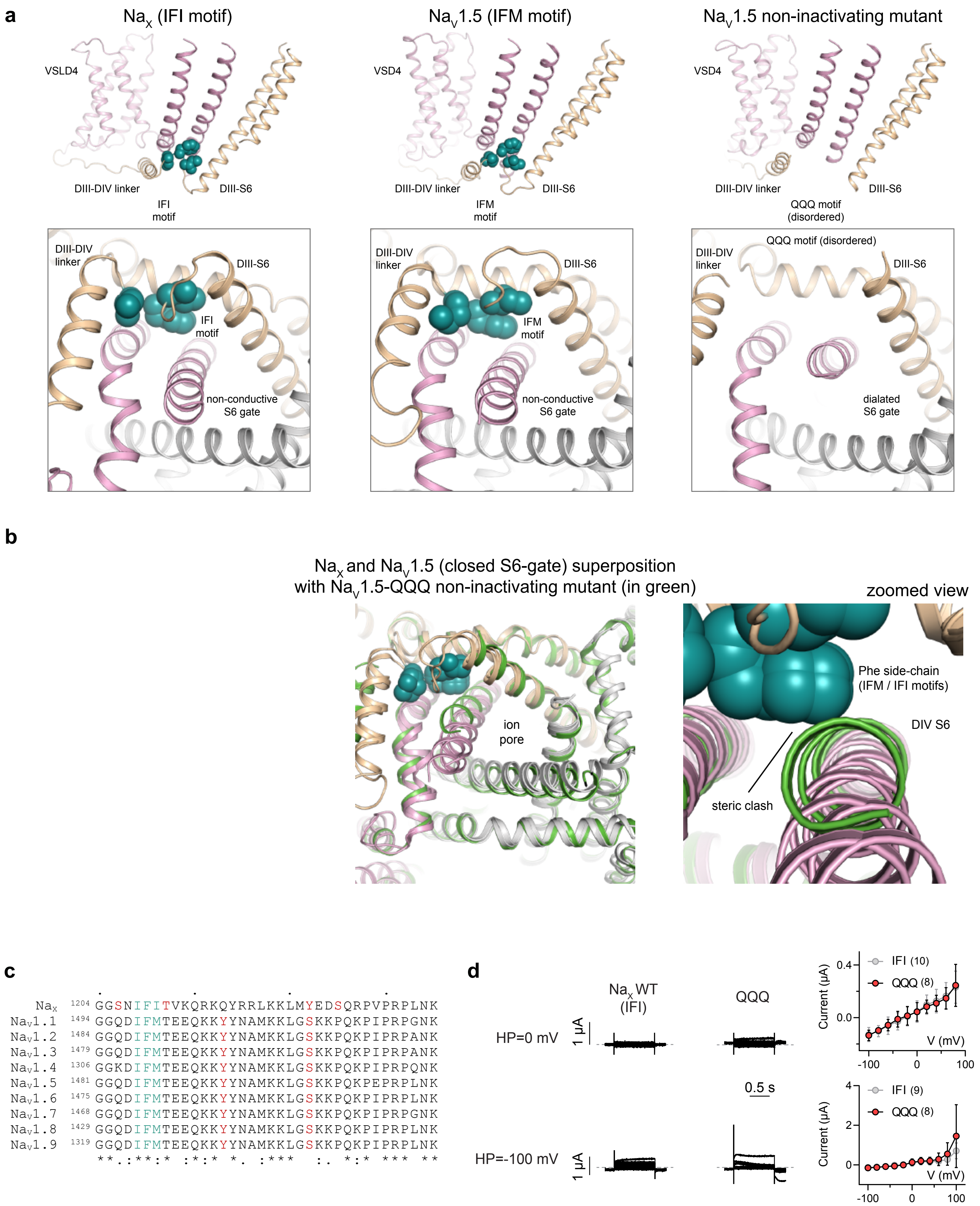
a
Nax (IFI motif)
Nav1.5 (IFM motif)
Nav1.5 non-inactivating mutant
VSLD4
VSD4
DIII-DIV linker
DIII-S6
IFI motif
IFM motif
QQQ motif (disordered)
non-conductive S6 gate
dialated S6 gate
b
Nax and Nav1.5 (closed S6-gate) superposition with Nav1.5-QQQ non-inactivating mutant (in green)
zoomed view
ion pore
Phe side-chain (IFM / IFI motifs)
DIV S6
steric clash
c
Nax 1204 GGSNIFITVKQRKQYRRLKKLMYEDSQRPVPRPLNK
Nav1.1 1494 GGQDIFMTEEQKKYYNAMKKLGSKKPQKPIPRPGNK
Nav1.2 1484 GGQDIFMTEEQKKYYNAMKKLGSKKPQKPIPRPANK
Nav1.3 1479 GGQDIFMTEEQKKYYNAMKKLGSKKPQKPIPRPANK
Nav1.4 1306 GGKDIFMTEEQKKYYNAMKKLGSKKPQKPIPRPQNK
Nav1.5 1481 GGQDIFMTEEQKKYYNAMKKLGSKKPQKPEPRPLNK
Nav1.6 1475 GGQDIFMTEEQKKYYNAMKKLGSKKPQKPIPRPLNK
Nav1.7 1468 GGQDIFMTEEQKKYYNAMKKLGSKKPQKPIPRPGNK
Nav1.8 1429 GGQDIFMTEEQKKYYNAMKKLGSKKPQKPIPRPLNK
Nav1.9 1319 GGQDIFMTEEQKKYYNAMKKLGSKKPQKPIPRPLNK
.::* :*:* *. :*** :. *:* *** **
d
Nax WT (IFI)
QQQ
HP=0 mV
HP=-100 mV
1 μA
0.5 s
IFI (10)
QQQ (8)
IFI (9)
QQQ (8)
Current (μA)
V (mV)
0.4
0.0
4
2
0
-100
100

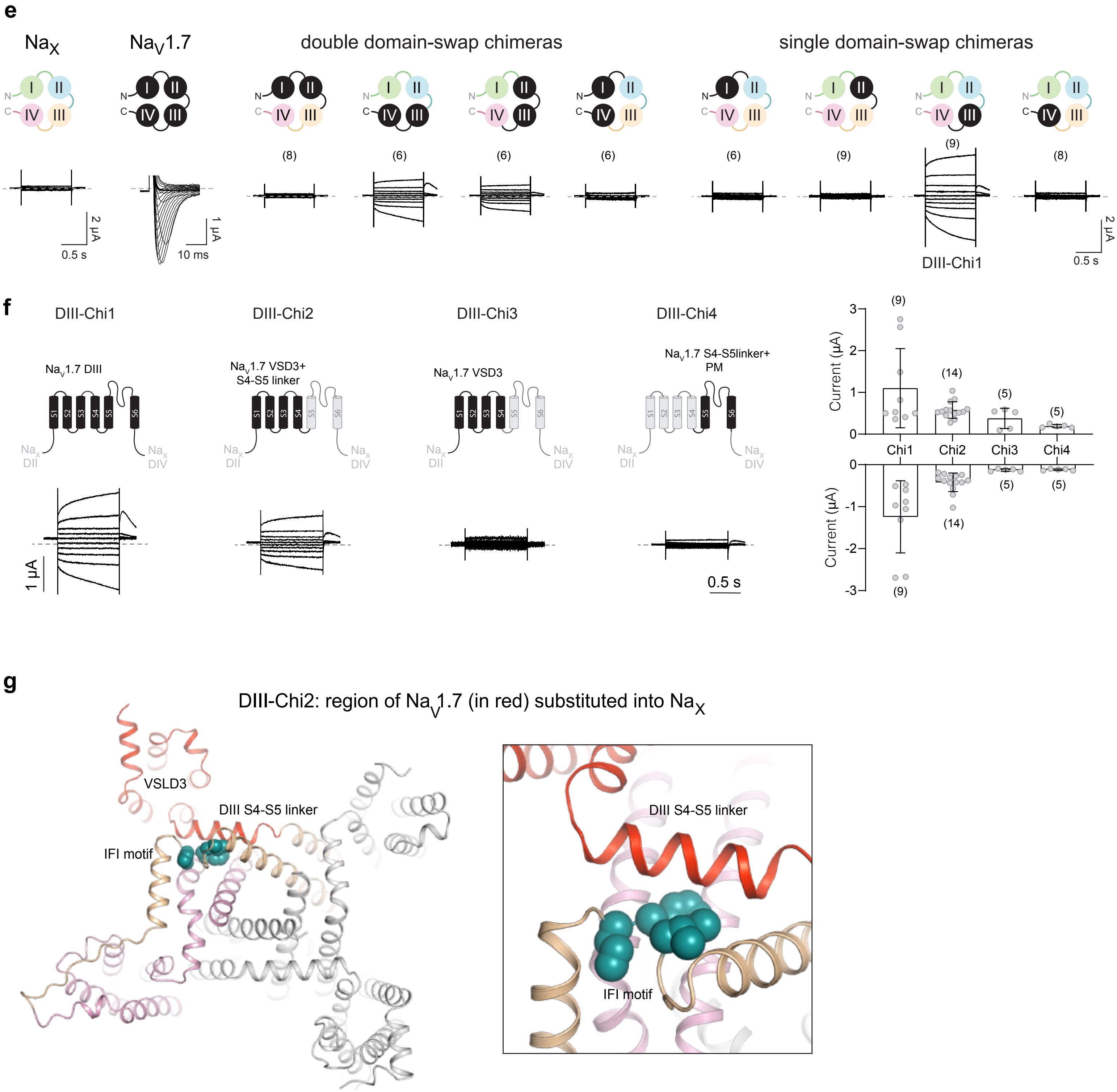

Supplementary Figure 7. Na_x DIII-DIV linker and $Na_v1.7$-Na_x chimeric channels.

a. Side- and intracellular views highlighting the DIII-DIV linker and IFI/IFM-motif (green spheres) of wild-type human Na_x, wild-type human $Na_v1.5$ (PDB 6LQA) and the rat $Na_v1.5$ non-fast inactivating IFM>QQQ motif mutant (PDB 7FBS) channels. Note, in these models, the Na_x pore is non-conductive (current study), wild-type $Na_v1.5$ is presumed to represent a non-conductive, inactivated-like state[26], and the IFM>QQQ motif mutant of rat $Na_v1.5$ is proposed to be in an open and conductive state[28]. **b.** Superposition of wild-type human Na_x, wild-type human $Na_v1.5$ (PDB 6LQA) and the rat $Na_v1.5$ non-fast inactivating IFM>QQQ motif mutant (PDB 7FBS) channels, with the QQQ mutant colored green. Zoomed view highlights the relative movement of the DIV S6 helix in rat $Na_v1.5$-QQQ, and the clash this would have with the IFM/IFI-motifs as positioned in wild-type human $Na_v1.5$ and Na_x, respectively. **c.** Multi-sequence alignment of the DIII-DIV linker region of human Na_x and Na_v channels. The IFI/IFM-motif is indicated in green and the known (Na_v channels) and NetPhos 3.1 server predicted (Na_x) phosphorylation sites are indicated in red. **d.** Representative currents from oocytes expressing wild-type or IFI>QQQ-mutant human Na_x with voltage steps between +80 and -100 mV, in 20 mV increments, from a HP of 0 mV, or depolarizing steps between -100 and +120 mV, in 20 mV increments, from a HP of -100 mV. I-V plots are shown on the right. Data are shown as mean ± SD. Numbers of biological replicates (*n*) are indicated. **e.** Schematics and representative currents from oocytes expressing various double-domain and single-domain swapped human $Na_v1.7$-Na_x channel chimeras in response to voltage steps between +80 and -100 mV, in 20 mV increments, from a HP of 0 mV. Numbers of biological replicates (*n*) are indicated. **f.** Schematics and representative currents from oocytes expressing focused DIII human $Na_v1.7$-Na_x channel chimeras in response to voltage steps between +80 and -100 mV, in 20 mV increments, from a HP of 0 mV. Right, maximal current amplitudes at +80 mV (top) and -100 mV (bottom) of the indicated DIII $Na_v1.7$-Na_x chimeras. Data are shown as mean ± SD. Numbers of biological replicates (*n*) are indicated. **g.** Intracellular view of Na_x with regions from human $Na_v1.7$ substituted in DIII-Chim2 highlighted in red.

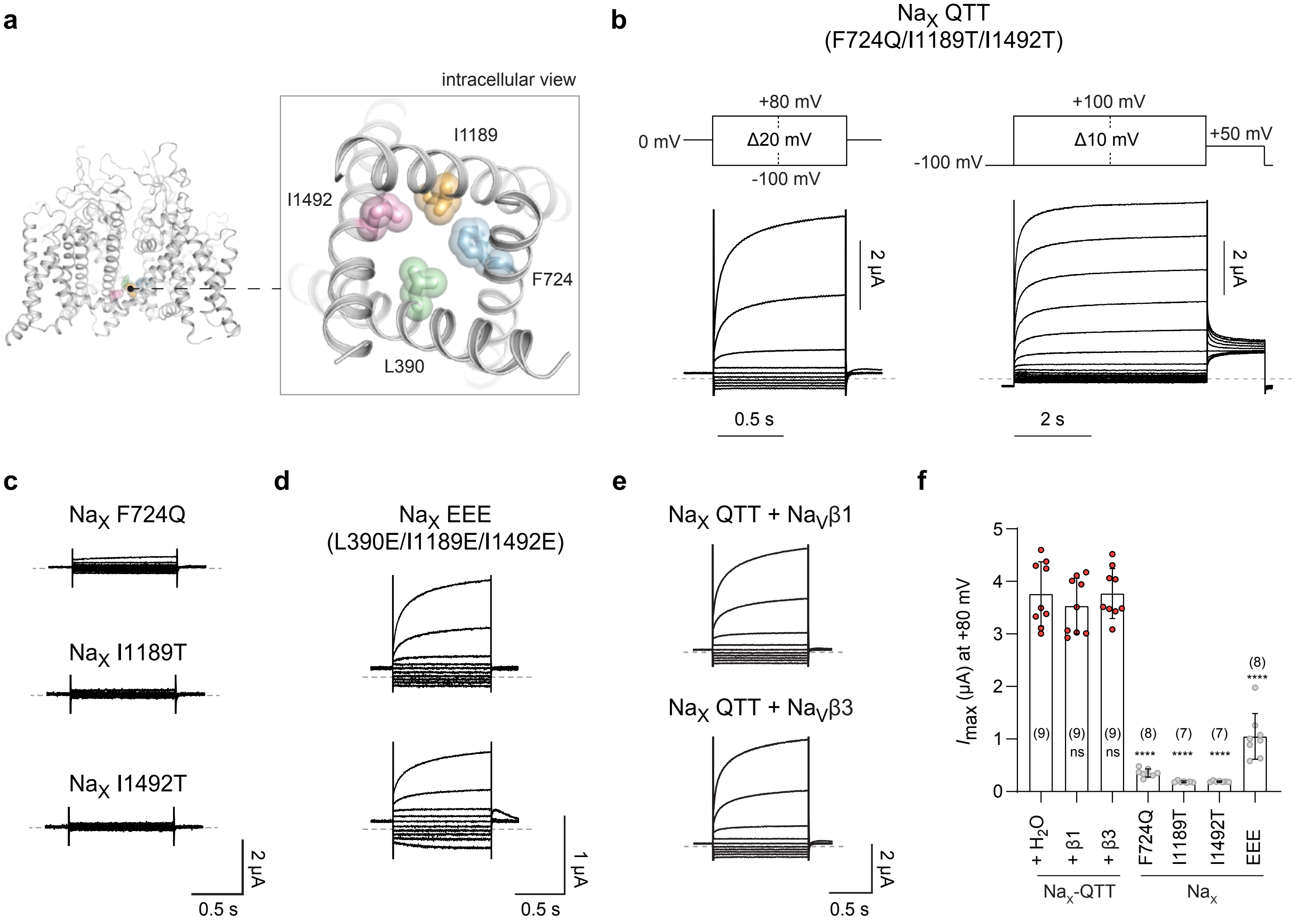

Supplementary Figure 8. Characterization of human Na_x carrying targeted pore-wetting S6-mutations (complementary and expanded Fig. 2e-g).

a. Location and zoomed view of targeted hydrophobic side-chains lining the S6-gate.

b. Representative currents from Xenopus laevis oocytes expressing the Na_x-QTT construct under indicated voltage protocols.

c. Representative currents from oocytes expressing the indicated Na_x construct. Voltage protocols as above.

d. Representative currents from oocytes expressing the Na_x-EEE construct. Voltage protocols as above.

e. Representative currents from oocytes expressing the Na_x-QTT construct and co-expressing indicated Na_v auxiliary subunit. Voltage protocols as above.

f. Data summary of independent experiments performed as in parts b-e. Data are shown as mean ± SD; ns not significant; ****p < 0.0001; one-way ANOVA with Dunnett's test (against Na_x-QTT+H_2O). Exact p values and statistical parameters are provided in Source Data. Numbers of biological replicates (*n*) are indicated.

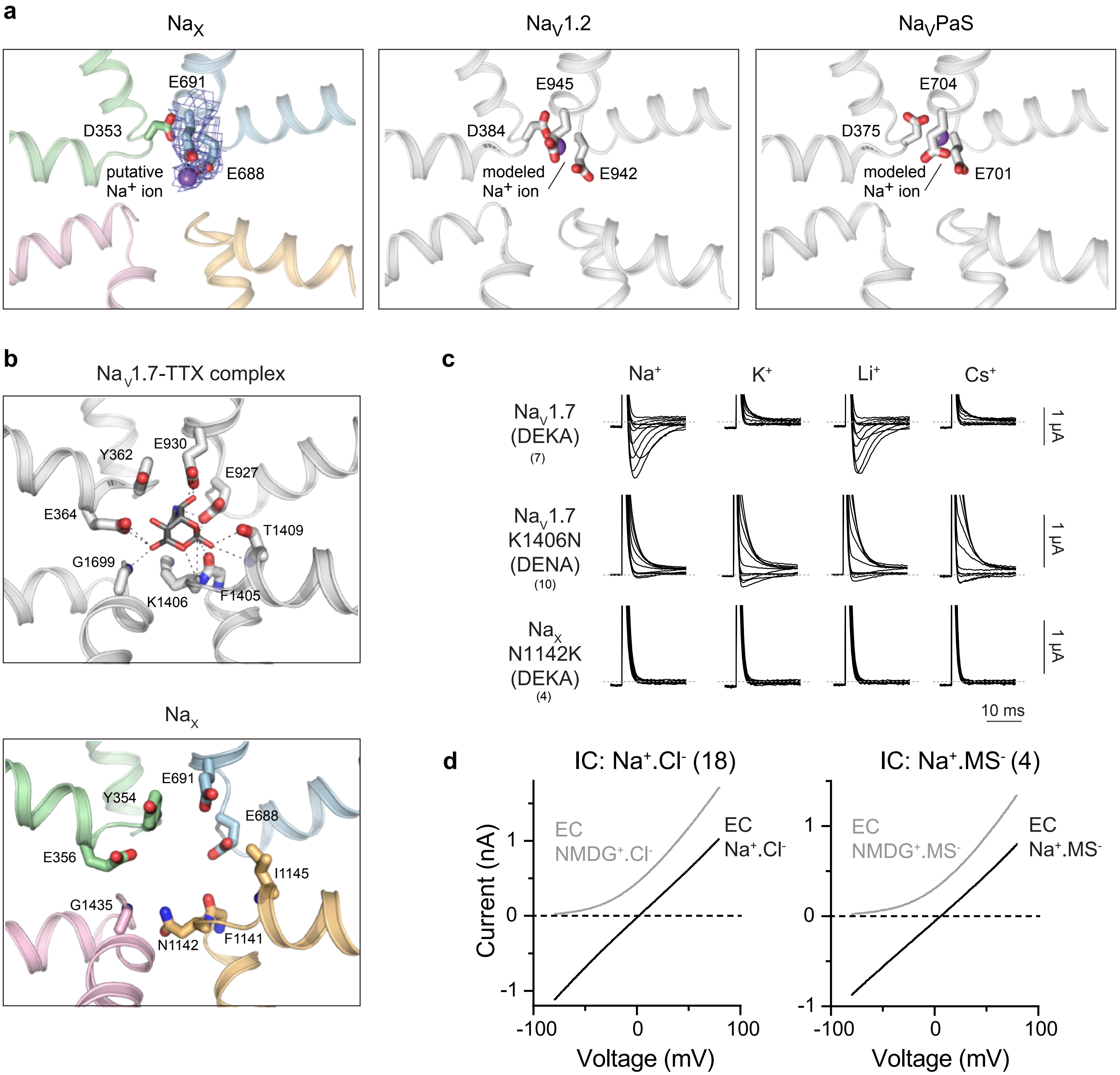

Supplementary Figure 9. Comparison of the Na_X and $Na_V1.7$ selectivity filters.
a. Close-up view of the Na_X selectivity filer (DEE motif shown in sticks) with a portion of the cryo-EM map (blue mesh) and a putative Na^+ ion[55] modeled as a purple sphere. This coordination site is consistent with a Na^+ ion binding site, but we cannot exclude the possibility this feature may be a water molecule or Ca^{2+} ion. The location of the assigned Na^+ ion (purple sphere) in the analogous DEE motif binding site in human $Na_V1.2$ (PDB 6J8E) and cockroach Na_VPaS (PDB 6NT4) channels are shown for comparison. **b.** Matched views of the NaX and $Na_V1.7$ (PDB 6J8J) selectivity filters. Tetrodotoxin is bound in $Na_V1.7$ and key interactions are shown; where most of these interacting groups are positionally conserved in the Na_X selectivity filter. **c.** Representative currents from *Xenopus laevis* oocytes expressing wild-type or mutant human $Na_V1.7$ and Na_X channel constructs in the presence of 115 mM extracellular Na^+, K^+, Li^+ or Cs^+. Steps between -50 and +50 mV, in 10 mV steps, from a HP of -100 mV are shown. Numbers of biological replicates (n) are indicated. **d.** Representative currents from HEK293 cells expressing human Na_X-QTT (containing a C-terminal GFP-Flag tag) in response to a voltage ramp from –80 to +80 mV in the presence (left) or absence (right) of chloride (Cl^-) ions (substituted with methanesulfonate, MS^-). Numbers of biological replicates (*n*) are indicated.

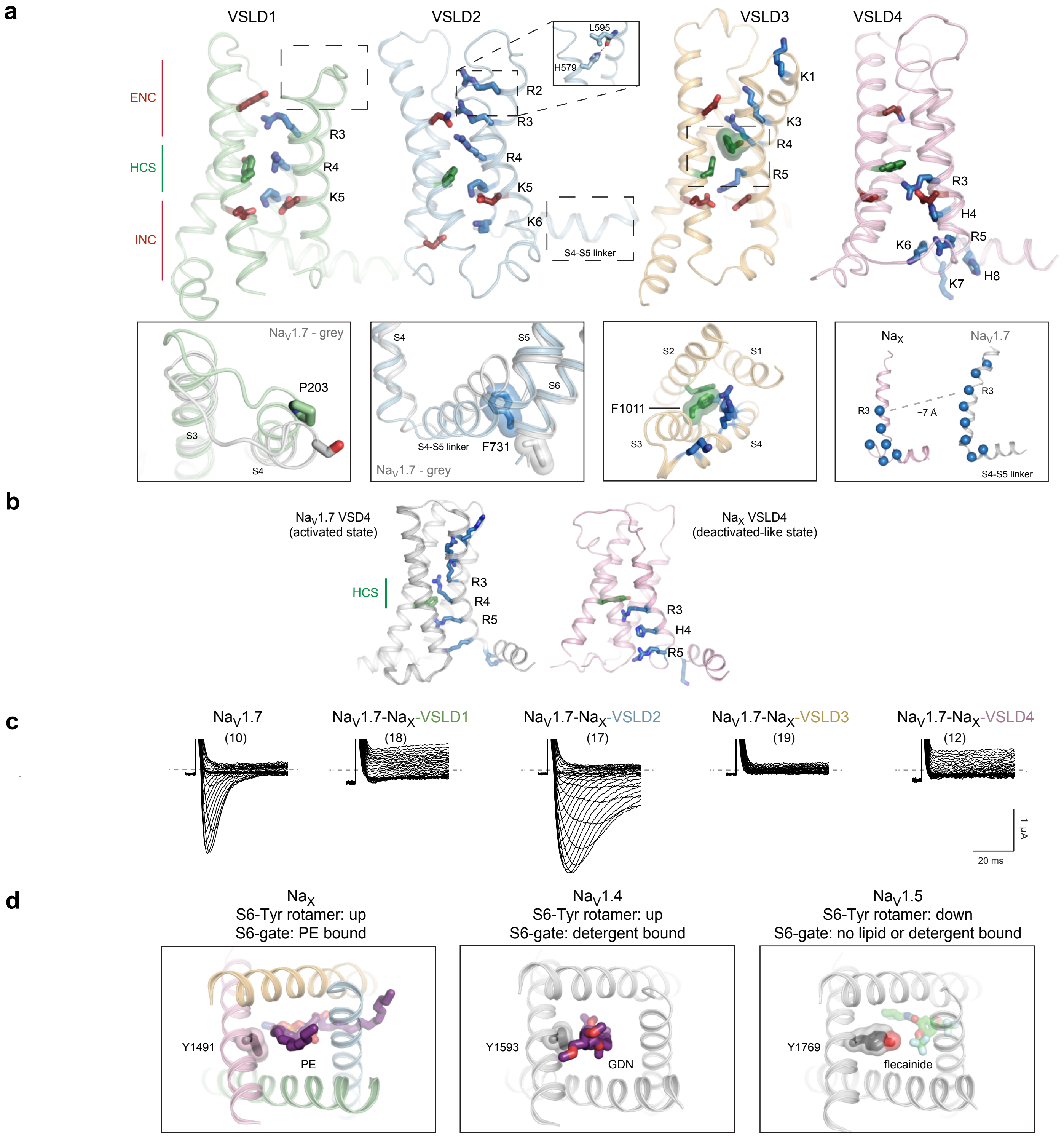

Supplementary Figure 10. Human Na_X has atypical voltage sensor-like domains but a common hydrophobic S6 gate.

a. VSLD1, VSLD2, VSLD3, and VSLD4 residues positionally equivalent to S4 gating charges (blue), the hydrophobic construction site (HCS, green), and the extracellular and intracellular negative charge clusters (ENC and INC, respectively, red) are shown in stick representation. VSLD1 insert highlights a unique proline in Na_X, a position conserved as serine in Na_V channels, where the Ser211Pro mutation in $Na_V1.7$ (PDB 6J8J) is reportedly pathogenic[56]. VSLD2 insets highlight a unique His579-S4 interaction (top) and S4-S5 linker displacements (bottom), where an analogous DII S6 sequence in $Na_V1.7$ (i.e. ΔLeu966, see Supplementary Fig. 2) is reportedly pathogenic[57]. VSLD3 inset highlights a unique phenylalanine (Phe1011, green surface) from the S3 helix which is conserved as a serine in Na_V and KCNQ1 channels, where the analogous S3 mutation (Ser209Phe) mutation in KCNQ1 is reportedly pathogenic[58]. VSLD4 inset shows a comparison of the Cα of gating charge residues (blue spheres) relative to $Na_V1.7$ VSD4 (PDB 6J8J). **b.** View of human $Na_V1.7$ VSD4 and Na_X VSLD4 highlighting the HCS (green) and positions equivalent to the S4 gating charges (blue). **c.** Representative currents from *Xenopus laevis* oocytes expressing $Na_V1.7$ and various $Na_V1.7$-Na_X-VSLD channel chimeras in response to depolarizing steps between -80 and +65 mV in 5 mV increments from a HP of -100 mV. Numbers of biological replicates (*n*) are indicated. **d.** Intracellular view of the Na_X S6-gate with Tyr1491 (S6) and bound phosphatidylethanolamine shown and compared to $Na_V1.4$ (assumed to be in a non-conductive, fast-inactivated state; PDB 6AGF) and drug-bound $Na_V1.5$ (assumed to be in a non-conductive, drug blocked inactivated state; PDB 6UZ0). Assigned lipid, detergent and drug molecules are show in purple (PE and GDN) and green (flecainide) stick representations, respectively.

Supplementary Table 1. Cryo-EM data collection, refinement and validation statistics

	β3-Na_X-nanodisc (EMDB-25919) (PDB 7TJ8)	β3-Na_X-GDN (EMDB-25920) (PDB 7TJ9)
**Data collection and processing**		
Magnification	165,000	105,000
Voltage (kV)	300	300
Electron exposure (e–/Å^2)	48.579	64.009
Defocus range (μm)	0.5-1.5	0.5-1.5
Pixel size (Å)	0.849	1.1648
Symmetry imposed	C1	C1
Initial particle images (no.)	1,968,741	2,780,663
Final particle images (no.)	1,238,338	1,420,422
Map resolution (Å)	3.2	2.9
FSC threshold	0.143	0.143
Map resolution range (Å)	3.2 – 46.6	2.9 – 33.13
**Refinement**		
Initial model used (PDB code)	6AGF	β3-Na_X-nanodisc
Model resolution (Å)	3.4	3.1
FSC threshold	0.5	0.5
Model resolution range (Å)	3.2 – 46.6	2.9 – 33.13
Map sharpening *B* factor (Å^2)	-90	-90
Model composition		
Non-hydrogen atoms	21502	22342
Protein residues	1242	1273
Ligands	18	23
B factors (Å^2)		
Protein	67.58	77.83
Ligand	64.16	70.41
R.m.s. deviations		
Bond lengths (Å)	0.004	0.006
Bond angles (°)	0.906	0.611
Validation		
MolProbity score	1.78	1.66
Clashscore	5.79	5.15
Poor rotamers (%)	0.80	0.09
Ramachandran plot		
Favored (%)	93.72	94.85
Allowed (%)	5.79	5.15
Disallowed (%)	0.49	0.00
