# Supplementary material for: Structure-guided unlocking of NaX reveals a non-selective tetrodotoxin-sensitive cation channel
Source: Nat Commun. 2022 Mar 17;13:1416. doi: 10.1038/s41467-022-28984-4 (PMC8931054; doi:10.1038/s41467-022-28984-4)
Supplement: Supplementary file 2 — Reporting Summary [file 41467_2022_28984_MOESM2_ESM.pdf]

Corresponding author(s): Claudio Ciferri, Stephan Pless, Jian Payandeh

Last updated by author(s): February 10th 2022

Reporting Summary

Nature Research wishes to improve the reproducibility of the work that we publish. This form provides structure for consistency and transparency in reporting. For further information on Nature Research policies, see our Editorial Policies and the Editorial Policy Checklist.

Statistics

For all statistical analyses, confirm that the following items are present in the figure legend, table legend, main text, or Methods section.

n/a	Confirmed	
☐	☒	The exact sample size (*n*) for each experimental group/condition, given as a discrete number and unit of measurement
☐	☒	A statement on whether measurements were taken from distinct samples or whether the same sample was measured repeatedly
☐	☒	The statistical test(s) used AND whether they are one- or two-sided<br>*Only common tests should be described solely by name; describe more complex techniques in the Methods section.*
☐	☒	A description of all covariates tested
☐	☒	A description of any assumptions or corrections, such as tests of normality and adjustment for multiple comparisons
☐	☒	A full description of the statistical parameters including central tendency (e.g. means) or other basic estimates (e.g. regression coefficient) AND variation (e.g. standard deviation) or associated estimates of uncertainty (e.g. confidence intervals)
☐	☒	For null hypothesis testing, the test statistic (e.g. *F*, *t*, *r*) with confidence intervals, effect sizes, degrees of freedom and *P* value noted<br>*Give P values as exact values whenever suitable.*
☒	☐	For Bayesian analysis, information on the choice of priors and Markov chain Monte Carlo settings
☒	☐	For hierarchical and complex designs, identification of the appropriate level for tests and full reporting of outcomes
☒	☐	Estimates of effect sizes (e.g. Cohen's *d*, Pearson's *r*), indicating how they were calculated

Our web collection on statistics for biologists contains articles on many of the points above.

Software and code

Policy information about availability of computer code

Data collection	Relion3.1.1, cisTEM, ChimeraX, UCSF Chimera, Carver3.0, MotionCor2, CTFFIND4.1, WARP, Phenix, Prism 9.3.1, Mascot, ISOLDE, pymol 2.5.2, pClamp 10.6.2, ESPript
Data analysis	All software and data analyses methods have been described and references appropriately cited

For manuscripts utilizing custom algorithms or software that are central to the research but not yet described in published literature, software must be made available to editors and reviewers. We strongly encourage code deposition in a community repository (e.g. GitHub). See the Nature Research guidelines for submitting code & software for further information.

Data

Policy information about availability of data

All manuscripts must include a data availability statement. This statement should provide the following information, where applicable:

- Accession codes, unique identifiers, or web links for publicly available datasets
- A list of figures that have associated raw data
- A description of any restrictions on data availability

all relevant data sets will be released from appropriate public data bases up publication, and are also available at any time upon request

Field-specific reporting

Please select the one below that is the best fit for your research. If you are not sure, read the appropriate sections before making your selection.

☒ Life sciences ☐ Behavioural & social sciences ☐ Ecological, evolutionary & environmental sciences

For a reference copy of the document with all sections, see nature.com/documents/nr-reporting-summary-flat.pdf

Life sciences study design

All studies must disclose on these points even when the disclosure is negative.

Sample size	No pre-determined sample size was estimated. All electrophysiological experiments were performed on a minimum of 3 cells and the exact numbers of recordings were chosen to ensure sufficient statistical power, while maintaining reasonable cost and workload. Under these conditions and in light of our p values, we are confident that our sample sizes are sufficient to determine differences between constructs and conditions. Biochemistry experiments were performed a minimum of 3 times and results were reproducible.
Data exclusions	Electrophysiological recordings with significant endogenous currents at highly de- or hyperpolarised potentials were not considered for analysis. Mock- or uninjected cells were recorded as controls for endogenous current levels for every batch. These were pre-established criteria.
Replication	Measurements from multiple cells were performed using multiple batches of transfections or oocyte isolations. The number of independent experiments performed are shown in the figures (or figure legends). All replication attempts were successful, incl from batch to batch.
Randomization	Randomization in electrophysiological recordings was not necessary because experimental groups were recorded in varying order (either WT vs different constructs or construct combinations, or constructs with or without compound)
Blinding	Blinding was not possible for this study because individuals prepared their own experiments. However, electrophysiological recordings were performed by different individuals and yielded consistent results.

Reporting for specific materials, systems and methods

We require information from authors about some types of materials, experimental systems and methods used in many studies. Here, indicate whether each material, system or method listed is relevant to your study. If you are not sure if a list item applies to your research, read the appropriate section before selecting a response.

Materials & experimental systems

n/a	Involved in the study	
☐	☒	Antibodies
☐	☒	Eukaryotic cell lines
☒	☐	Palaeontology and archaeology
☐	☒	Animals and other organisms
☒	☐	Human research participants
☒	☐	Clinical data
☒	☐	Dual use research of concern

Methods

n/a	Involved in the study	
☒	☐	ChIP-seq
☒	☐	Flow cytometry
☒	☐	MRI-based neuroimaging

Antibodies

Antibodies used	Mouse anti-beta-actin 1:1000 (sc-47778 SantaCruz Biotechnologies), mouse anti-Na+/K+-ATPase alpha1 C464.6 1:2000 (sc-21712 SantaCruz Biotechnologies), rabbit anti-FLAG 1:2000 (701629 Invitrogen), IRDye680 goat anti-mouse 1:5000 (926-68070 LI-COR Biosciences), IRDye800 goat anti-rabbit 1:5000 (926-32211 LI-COR Biosciences)
Validation	All antibodies were tested against appropriate controls performed and have also been reported extensively in the literature [e.g. PMID: 31013932 (anti-beta-actin), PMID: 31914671 (anti-Na/K ATPase)]

Eukaryotic cell lines

Policy information about cell lines

Cell line source(s)	HEK293T (human embryonic kidney cells) and mouse Neuro-2a cells (CCL-131) purchased from ATCC. Expi293 cells were purchased from ThermoFisher Scientific.
Authentication	The cell line was not authenticated.

Mycoplasma contamination	The HEK293T and Neuro-2a cells were tested negative for mycoplasma contamination.
Commonly misidentified lines (See ICLAC register)	No commonly misidentified cell lines were used

Animals and other organisms

Policy information about studies involving animals; ARRIVE guidelines recommended for reporting animal research

Laboratory animals	Female Xenopus laevis oocytes
Wild animals	No wild animals were used in this study
Field-collected samples	n/a
Ethics oversight	Stage V/VI oocytes were obtained from ovaries of female Xenopus laevis frogs (obtained from African Reptile Park, South Africa). Animals were anaesthetized in 0.3% tricaine. Animal work and handling was carried out under license 2014–15-0201–00031, approved by the Danish Veterinary and Food Administration. Frogs were housed and cared for by an animal facility with ethical approval from the University of Copenhagen, Denmark.

Note that full information on the approval of the study protocol must also be provided in the manuscript.
